# Nucleus Accumbens Corticotropin-Releasing Hormone Neurons Projecting to the Bed Nucleus of the Stria Terminalis Promote Wakefulness and Positive Affective State

**DOI:** 10.1007/s12264-024-01233-y

**Published:** 2024-07-09

**Authors:** Gaojie Pan, Bing Zhao, Mutian Zhang, Yanan Guo, Yuhua Yan, Dan Dai, Xiaoxi Zhang, Hui Yang, Jinfei Ni, Zhili Huang, Xia Li, Shumin Duan

**Affiliations:** 1 Institute for Translational Brain Research, MOE Frontiers Center for Brain Science, Fudan University, Shanghai, 200032 China; 2 Department of Pharmacology, School of Basic Medical Sciences, State Key Laboratory of Medical Neurobiology, Institutes of Brain Science and Collaborative Innovation Center for Brain Science, and Joint International Research Laboratory of Sleep, Fudan University, Shanghai, 200032 China; 3 Institute of Special Environmental Medicine, Co-innovation Center of Neuroregeneration, Nantong University, Nantong, 226000 China; 4 Neurovascular Center, Changhai Hospital, Naval Medical University, Shanghai, 200433 China; 5 Department of Neurosurgery, Huashan Hospital, Fudan University, Shanghai, 200040 China; 6 State Key Laboratory of Medical Neurobiology and MOE Frontiers Center for Brain Science, Shanghai Medical College, Fudan University, Shanghai, 200032 China; 7 NHC and CAMS Key Laboratory of Medical Neurobiology, MOE Frontier Science Center for Brain Science and Brain-Machine Integration, School of Brain Science and Brain Medicine, Zhejiang University, Hangzhou, 310030 China

**Keywords:** Corticotropin-releasing hormone, Wakefulness, Anxiolysis, Nucleus accumbens, Bed nucleus of the stria terminalis

## Abstract

The nucleus accumbens (NAc) plays an important role in various emotional and motivational behaviors that rely on heightened wakefulness. However, the neural mechanisms underlying the relationship between arousal and emotion regulation in NAc remain unclear. Here, we investigated the roles of a specific subset of inhibitory corticotropin-releasing hormone neurons in the NAc (NAc^CRH^) in regulating arousal and emotional behaviors in mice. We found an increased activity of NAc^CRH^ neurons during wakefulness and rewarding stimulation. Activation of NAc^CRH^ neurons converts NREM or REM sleep to wakefulness, while inhibition of these neurons attenuates wakefulness. Remarkably, activation of NAc^CRH^ neurons induces a place preference response (PPR) and decreased basal anxiety level, whereas their inactivation induces a place aversion response and anxious state. NAc^CRH^ neurons are identified as the major NAc projection neurons to the bed nucleus of the stria terminalis (BNST). Furthermore, activation of the NAc^CRH^-BNST pathway similarly induced wakefulness and positive emotional behaviors. Taken together, we identified a basal forebrain CRH pathway that promotes the arousal associated with positive affective states.

**Supplementary Information:**

The online version contains supplementary material available at 10.1007/s12264-024-01233-y.

## Introduction

Nucleus accumbens (NAc) is critical for mediating motivation, reward, and a diverse range of stress responses that depend on heightened arousal [1–4]. Recent studies indicate that the NAc regulates sleep-wake behaviors through specific pathways. Activation of NAc dopamine D1 receptor (D1R)-expressing neurons (direct pathway) projecting to the midbrain and lateral hypothalamus has been reported to promote wakefulness [5], whereas activation of NAc dopamine D2 receptor (D2R)-expressing neurons (indirect pathway) project to the ventral pallidum induces non-rapid eye movement (NREM) sleep [6]. These distinct subtypes of GABAergic projection neurons also exhibit complementary or even opposing roles in other behaviors. For example, activation of D1R-positive neurons primarily elicits reward and reinforcement effects [7], whereas activation of D2R-positive neurons is associated with aversion response [8]. Given the diverse subtypes of neurons within the NAc projecting to distinct brain areas involved in various functions, it is imperative to elucidate the roles and the underlying circuit mechanisms of specific neuronal subpopulations of NAc in regulating sleep-wake and emotional behaviors.

Corticotropin-releasing hormone (CRH) has been recognized as a critical stress-related neuroendocrine signaling in the hypothalamic-pituitary-adrenal axis [9–12]. However, CRH-positive neurons also distribute in other brain regions and may regulate brain functions through specific neural circuits [13, 14]. Anatomical studies have revealed that NAc is enriched with CRH-positive neurons and fibers [15]. Furthermore, CRH in NAc has been reported to regulate a variety of brain functions [16–19]. However, most of these studies relied on pharmacological evidence that may reflect the effects of CRH inputs from other brain regions projecting to NAc neurons [15, 20, 21], the functional roles of NAc^CRH^ neurons and the underlying circuit mechanisms remain unclear.

In the present study, we identified that NAc^CRH^ neurons expressing DR1 constitute the major projecting pathway to BNST. Optogenetic and pharmacogenetic manipulation of NAc^CRH^ neurons or their projection terminals in BNST robustly modulate arousal levels and emotional behaviors.

## Materials and Methods

### Animals

CRH-Cre mice (B6(Cg)-Crh^tm1(cre)Zjh^/J, the Jackson Laboratory, Stock No: 012704) were housed (3–5 per cage) under an automatic 12 h (07:00–19:00) light/dark cycle within a soundproof room (22 ± 1 °C temperature, 60% ± 2% humidity, food and water *adlibitum*) [22]. All experimental protocols were approved by the Animal Care and Use Committee of Fudan University. All procedures were carried out following the Guidelines of the NIH (United States) regarding the care and use of animals.

### Preparation of Adeno-associated Viruses (AAVs)

The adeno-associated virus (AAV) vectors, namely, AAV-hSyn-DIO-hM3Dq-mCherry (Brain VTA, # PT-0042), AAV-hSyn-DIO-hM4Di-mCherry (Brain VTA, #PT-0043), AAV-hSyn-DIO-mCherry (Brain VTA, # PT-0013), AAV-hSyn-DIO-ChR2-mCherry (Brain VTA, #PT-0002), AAV-DIO-mGFP-T2A-Synaptophysin-mRuby (Taitool Bioscience, #S0314-4), AAV-EF1α-DIO-GCamp7f (Taitool Bioscience, #S0317-9), were packaged into the 2/9 AAV serotype and titrated at 3–5 × 10^12^ genome copies/mL.

### Surgeries and Viral Injections

Adult mice (8–12 weeks old) were anesthetized with isoflurane (5% induction, 1.5% ± 0.5% maintenance) and were then placed on a stereotaxic apparatus (RWD Life Science, China). Throughout the experiment, each mouse was heated using a heating pad. After asepsis, an incision was made to expose the skull, and remove the overlying connective tissue, and small craniotomy holes above the superficial layer of the NAc were made for viral injections. Through a fine glass pipette AAVs were slowly bilaterally microinjected (25 nL/min, the total volume of AAV the authors injected was 70 nL) into the NAc (anteroposterior (AP) = + 1.3 mm, mediolateral (ML) = ± 1.1 mm, dorsoventral (DV) = − 4.1 mm). For the tracer retrograde labeling of NAc^CRH^-BNST, CTB Alexa Fluor 647 (Thermo Fisher Scientific, Massachussetts, USA. 100 nL per side, 1 μg/μL) was injected into the BNST (AP = + (0.1–0.3) mm, ML = ± (0.8–0.9) mm, DV = − (4.7– 4.75) mm). The glass pipette was left in place for 10 min and was then slowly withdrawn. Three weeks after AAV injections, the mice used for* in vivo* optogenetic stimulation studies were bilaterally implanted with EEG/EMG electrodes and fibers (125 µm outer diameter (OD), 0.37 numerical aperture (NA), Newdoon, Hangzhou, China) above the NAc (AP = + 1.3 mm, ML = ± 1.1mm, DV = − 4.0 mm), or BNST (AP = + (0.1–0.3) mm, ML = ± (0.8–0.9) mm, DV = − (4.6–4.65) mm). For cannula guide (model 62059, RWD Life Science) implantation, the coordinates (AP = + (0.1–0.3) mm, ML = ± (0.8–0.9) mm, DV = − (4.6–4.65) mm) were used to target BNST bilaterally. Dental cement was used to secure the cannulas to the skull. After implantation, A heating pad was used to recover the mice. Mice were allowed housed for at least 1–2 weeks after optical fiber implantation or cannula guide implantation for complete recovery before experiments. For the intracranial infusion of CNO experiment, mice anesthetized with isoflurane and were assigned to receive bilateral 300 nL/side injections of CNO (1 mmol/L) or saline with a hydraulic pump (model R462, RWD Life Science) at a speed of 40 nL per minute. Viral expression was verified by the sacrifice of mice after behavioral testing.

### *In vivo* Fiber Photometry Recording and Data Analysis

As previously described [23], briefly, fluorescent emission was recorded with a fiber photometry system (Thinker Tech Nanjing Bioscience Inc.). To record the fluorescence of GCaMP, a 488 nm laser beam (OBIS 488LS, Coherent, USA) was reflected on a dichroic mirror (MD498, Thorlabs), then by using an objective 10 × lens (NA = 0.3, Olympus), a commutator has been coupled to the lens (Doric Lenses, Canada) to facilitate focus. Between the commutator and the implanted optical fiber, an optical fiber (230 mm O.D., NA = 0.37, 1 m long) guided the light. To minimize bleaching at the tip of the optical fiber, the laser power was adjusted to a low level of 10–20 μW. Photomultiplier tubes (R3896, Hamamatsu) were used to collect GCaMP fluorescence after it was bandpass filtered (MF525-39, Thorlabs). Photomultiplier tube current signals were converted to voltage signals using an amplifier (C7319, Hamamatsu), and then filtered using a low-pass filter (Brownlee 440, 40 Hz cut-off). The mice were placed in the recording homecage for two days in advance, then conducted fiber photometry experiments both in the light and dark periods. A Power1401 digitizer and Spike 2 software (CED, Cambridge, UK) were used to downsample the photometry voltage traces and interpolation to match the EEG/EMG sampling rate of 512 Hz. The resultant signal was analyzed using custom MATLAB code.

Briefly, polysomnographic photometry data were exported to MATLAB Mat files from Spike 2. We derived the value of the photometry signal (∆*F*/*F*) by calculating (*F*-*F*0)/*F*0, where *F*0 is the mean fluorescence signal. Data were recorded for 4–6 h per mouse for sleep-wake analysis, and the averaged ∆*F*/*F* during all vigilance states was calculated. To analyze the state transition, we determined each transition in the data and aligned ∆*F*/*F* in a ± 50 s window around each point. Photometry data were analyzed with custom-written MATLAB codes (MATLAB R2019b, MathWorks).

### Optogenetic Stimulation During Polygraphic Recordings

For* in vivo* light stimulation, we performed optogenetic experiments between 8:00 a.m.–8:00 p.m. For acute photostimulation activation of NAc^CRH^ neurons or their axonal terminals, each trial consisted of 5 ms pulses of various frequencies lasting for 20 s using a 473 nm blue laser conducted (5–7 mW at the fiber tip, Shanghai Optogenetic stimulation* in vivo* Laser, China). For the chronic photostimulation procedure, programmed light-pulse trains (5 ms pulses at 20 Hz for 10-s on and 20-s off for 120 cycles) were used from 10:00 a.m.–11:00 a.m. Optogenetic stimulation was conducted over a 10-minute interval between trials. EEG/EMG recordings during the same period on the previous day served as a baseline control. A calculated sleep-wake cycle was obtained by scoring the whole hour offline for each animal.

### FISH by RNAscope

Mice were intracardially perfused with 0.9% saline followed by 4% paraformaldehyde in PBS. Brains were removed and fixed in 4% PFA buffer at 4°C overnight, and dehydrated in 30% sucrose until sinking. Brains were sectioned into about 10 μm coronal sections containing the NAc via a freezing microtome (Leica CM 1950, Germany) and collected into DepC-PBS. Following the RNAscope procedures (Advanced Cell Diagnostics, Inc., Newark, CA, USA). In brief, sections were thaw-mounted on slides and heated at 60 °C for 35 min then kept at − 80 °C before the experiment. Subsequently, the slides were treated once with 4% PFA (5 min) and thrice with ethanol (50%, 75%, 100%, 5 min each time) and air dried at room temperature. Next, the slides were pretreated for hydrogen peroxide for 10 min at room temperature and washed twice in DepC-PBS (2 min each time). The protease digestion was performed in a 40°C HybEZ oven for 30 min and washed twice in DepC-PBS (3 min each time) then rinsed in DepC-ddH_2_O. After that, the slides were hybridized with pre-warmed *Drd1* probe, *Drd2* probe, *Crh* probe, *Slc32a1* probe (VGAT), negative control probe (dihydrodipicolinate reductase (dapB) gene) or positive control probe (peptidylprolyl isomerase B, Ppib) in the 40 °C HybEZ oven for 2 h. Washing the slides in 1× washing buffer at room temperature to remove the nonspecifically hybridized probe, then followed by Amplifier 1-FL for 30 min, Amplifier 2-FL for 30 min, and Amplifier 3-FL for 15 min at 40 °C. Each amplifier was removed by washing with 1× washing buffer for 3 min at room temperature. At least eight brain slices from each mouse were performed in RNAscope and imaged. Poorly stained slides were not analyzed.

### Slice Preparation and Whole-Cell Recordings

Acute coronal brain slices were cut on a vibratome in ice-cold oxygenated cutting solution (in mmol/L): 215 sucrose, 10 D-glucose, 26 NaHCO_3_, 3 MgSO_4_, 2.5 KCl, 1.25 NaH_2_PO4, 0.4 ascorbic acid, 0.6 mmol/L Na-pyruvate, and 0.1 CaCl_2_, pH 7.4, 300 mOsm. Next, slices were transferred to a holding chamber and allowed to recover for 35 min at 32 °C and then maintained at room temperature (RT) in normal recording aCSF (in mmol/L: 126 NaCl, 2.5 KCl, 2.4 CaCl_2_, 26 NaHCO_3_, 25 glucose, 1.25 NaH_2_PO_4_ and 1.0 MgSO_4_). Patch pipettes (3–5 MΩ) for whole-cell recording were filled with an internal solution containing (in mmol/L): 130 CsCl, 0.5 CaCl_2_, 1 EGTA, 2 MgCl_2_, 2 QX-314, 0.4 GTP-Na, 2 ATP-Na, pH 7.3, 285–290 mOsm. During recordings, slices were placed on a glass coverslip and immersed in a recording chamber superfused with aCSF (2.5 mL/min), which were performed at 32°C using a heater controller (TC-324C, Warner Instruments). Expression of ChR2 was confirmed by visualization of bright mCherry fluorescence in CRH-expressing neurons and axon terminals. Whole-cell recordings were amplified with Multiclamp 700 B (filtered at 2.2 kHz), digitized with Digidata 1440 A (10 kHz), and recorded using pClamp 10.5 software (RRID: SCR_011323). Series resistance was monitored and the values were generally <10 MΩ and were not compensated. NAc^CRH^-ChR2- expressing neurons, axons, and synaptic terminals responses were evoked by 5 ms flashes of blue light (473 nm, 20 Hz, 3–5 mW) delivered from a single-wavelength laser system (Newdoon Inc., Hangzhou, China). IPSC signals were recorded at −70 mV in aCSF containing tetrodotoxin (TTX, 1 μmol/L), followed by a combination of TTX and 4-aminopyridine (4-AP, 100 μmol/L), and finally by a combination of TTX, 4-AP, and picrotoxin (PTX, 100 μmol/L). Series resistance (Rs) compensation was not used and cells with Rs changes over 20% were discarded. Data were analyzed using the Igor Pro software (WaveMetrics). Off-line analysis was performed by averaging 5–8 traces. For chemogenetic recordings, we recorded the baseline level and added 10 μmol/L CNO to record hM3Dq-expressing neurons or hM4Di-expressing neurons in the NAc.

### Behavioral Tests

Mice were transferred to the testing room on the day of the test and acclimated to the room conditions for at least one hour. Each test session was followed by a thorough cleaning with 75% alcohol to remove any traces or odors left behind by the previous test. Mice were recorded and counted via a video tracking system (Logitech web camera) and the ANY-maze software 7.2.0.

#### Real-Time Place Preference (RTPP) Test

Mice were placed in the center of a 50 cm × 30 cm × 40 cm two-chamber apparatus with distinct striped patterns. Each mouse was allowed to explore both chambers without light stimulation on the pre-test day (30 min). on test day (30 min), once mice entered the assigned side chamber blue light stimulation (5 ms pulse width at 20 Hz, ~5 mW) was delivered, otherwise turned off. On the post-test day (30 min), mice were allowed to freely explore both chambers without blue light stimulation.

#### Open Field Test

Mice were placed in an open field chamber (50 cm × 50 cm × 60 cm). The 15 min session was divided into three 5 min epochs when mice were tested. First epoch: there was no light stimulation (light-off), second epoch: animals received light stimulation (5 ms pulse width at 20 Hz, ~5 mW for NAc^CRH^ neurons or their axonal terminals), third epoch: there was no light stimulation (light-off). Blue laser light was delivered bilaterally during the light-on phase.

#### Elevated Plus Maze Test

The maze apparatus consists of two opposing open arms and two enclosed arms (35 cm × 6 cm) extending from a central platform (6 cm × 6 cm × 6 cm) at 90 degrees in the form of a plus sign. The maze was raised 70 cm above the floor. Mice were placed in the center platform and allowed to freely explore for 10 min. For the test photostimulation procedure, programmed light-pulse trains (5 ms pulses at 20 Hz for 10-s on and 20-s off for 20 cycles) were conducted.

#### Conditioned Place Aversion (CPA) Test

Mice were habituated to the chamber on day 1 (baseline, 30 min). On days 2–6, Mice were confined to the assigned chamber with CNO (3 mg/kg, i.p.) or saline injection (i.p.) for 30 min. On day 7, Mice was allowed to explore both chambers. Location plots and total time of the mice were recorded and counted.

#### Response to Chocolate Pellet Consumption

Before recording day, mice were food-deprived for 24 h and then habituated for 30 min to the environment. During testing, the mice were then habituated to a dimly lit (45 lux) rectangular chamber (20 cm × 20 cm × 20 cm, L × W × H, Shanghai ShiBo Intelligent Technology Co., Ltd) and with a video capture system, 35 min per day for 3 days. Allowing the mice freely exposed to food and water, a dish of chocolate pellets was introduced into one corner of the chamber. Then the mice were introduced to the chamber, and while eating chocolate pellets, they were subjected to photometry recordings. The fluorescence signals and mouse behaviors were captured simultaneously. The first three to five times that each mouse consumed were observed for fluorescence signals.

#### Response to Opposite-Sex Social Interaction

Before recording day, the mice were housed individually for at least 2–3 weeks. During testing, the mouse was placed in a rectangular acrylic chamber (40 cm × 40 cm × 40 cm, Shanghai ShiBo Intelligent Technology Co., Ltd) and with a video capture system, 30–60 min per day for 3 days. After the male-female interaction sessions, mice were exposed to the chamber for 10 min, then we introduced a female stranger mouse (6–8 weeks old) with sexual experience into the chamber of the test male mouse. Sniffing or mounting lasting at least three seconds was characterized as the onset of interaction. The fluorescence signals and mouse behaviors were captured simultaneously. The term “sniffing female” refers to when the male's nose is close to the female's face or body. An analysis of fluorescence signals was conducted while the female mouse sniffed.

#### Response to Sucrose Licking

Before recording day, mice were water-deprived for 12 hours and habituated for 30 minutes to the environment. Delivery of sucrose (2.5% w/v) was controlled by an inverted water bottle. A random interval duration of 20 to 40 seconds was set between sucrose trials. Then the mice were introduced into sucrose-licking chambers. A lick of the waterspout delivered 20 μL water followed by a 10-second timeout. Each licking behavior was tagged with a triggering TTL signal (Thinker Tech Nanjing Biotech.), which was synchronized with the fiber photometry. Fiber photometry was used to simultaneously record TTL signals and fluorescence signals. The fluorescence signals and mouse behaviors were captured simultaneously. The first five times that each mouse licking sucrose behavior was observed for fluorescence signals.

### Statistical Analysis

All experiments and data analysis were performed blinded, including the in vivo fiber photometry, electroencephalography (EEG)/electromyography (EMG), chemogenetic or optogenetic manipulations, electrophysiology, and behavioral. Data analysis was performed with GraphPad Prism v.8.0, MATLAB R2019b, Olympus FV10-ASW 4.0a Viewer, Image J-win64, Adobe Illustrator CS6, Adobe Photoshop CC 2018, and Microsoft Office 2019.

Data are presented as mean ± standard error of the mean (SEM). Data were assessed using one-way ANOVAs or Two-way ANOVAs to compare more than two groups or to perform group comparisons with multiple measurements. Paired or unpaired t-tests were used for comparisons between the two groups. All statistics were two-tailed tests. Statistical significance was set at *P* < 0.05 was considered statistically significant.

## Results

### Elevated Activities of NAc^CRH^ Neurons During Wakefulness

**Fig. 1:**
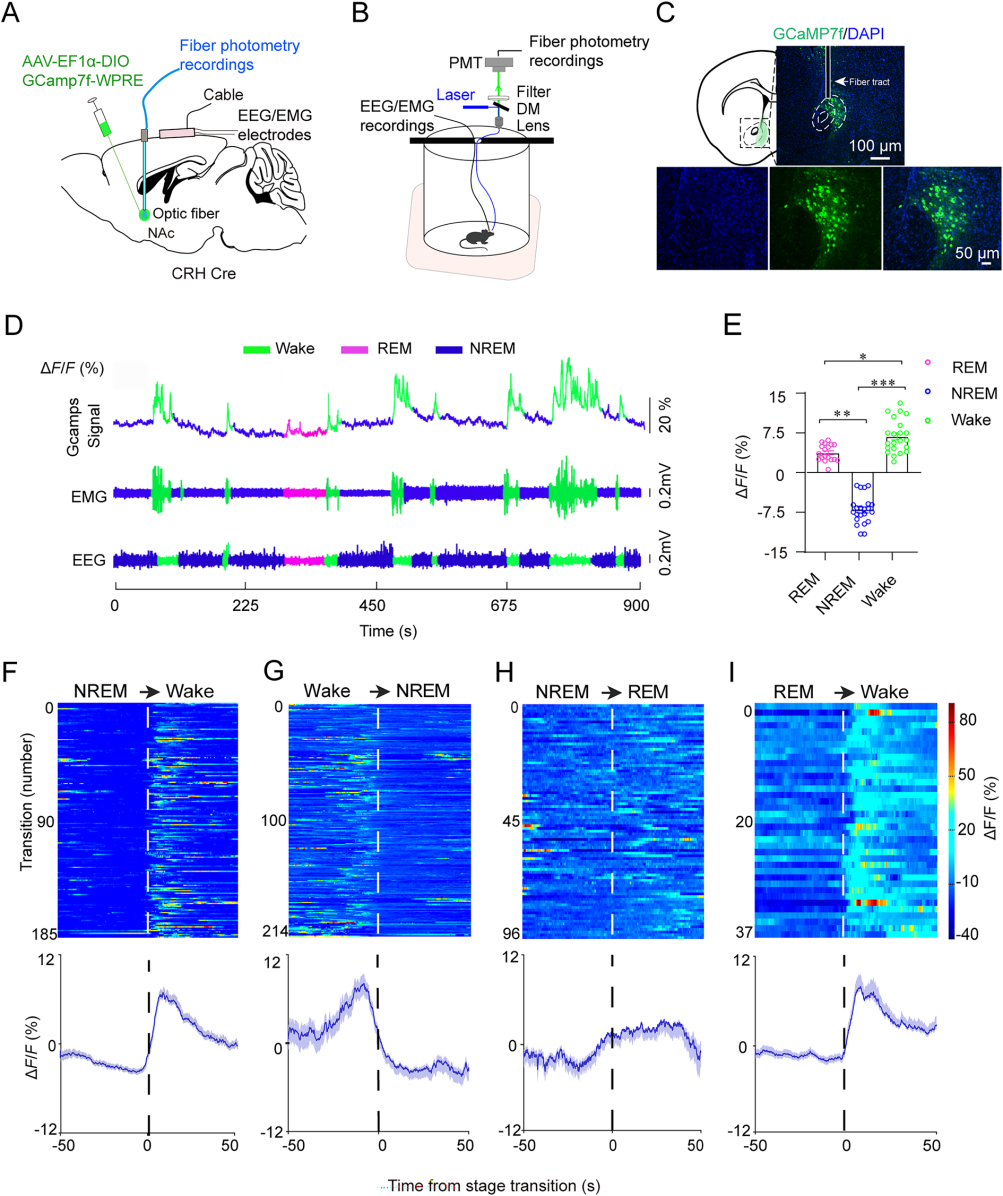
Population Ca^2+^ activities of NAc^CRH^ neurons across sleep-wake states. **A** and **B** Schematic of* in vivo* recording setup and AAV-EF1α-DIO-GCamp7f injection into the NAc of CRH-Cre mice. **C** Example images from a NAc brain section showing GCaMP7f expression (green) in CRH neurons in CRH Cre mouse. Blue, immunostaining for DAPI. Scale bar, 100 μm or 50 μm. **D** Representative raw traces of changes in Ca^2+^ activities (GCaMP7f fluorescence intensity) and EEG/EMG signals across spontaneous sleep-wake states. **E** Mean normalized fluorescence (∆*F/F*) during wake, REM sleep, and NREM sleep (*n =* 4 mice, one-way ANOVA between each state, followed by Holm-Sidak’s multiple comparisons test, *F*_2,52_ = 16.92, *P* = 0.0005, *P*(Wake-NREM) = 3 × 10^−5^,* P*(REM-Wake) = 6 × 10^−7^, *P*(NREM-REM) = 5 × 10^−6^). **F**–**I** Population Ca^2+^ activities of NAc^CRH^ neurons across state transitions. Upper panel: individual transitions with color-coded fluorescent intensities (NREM to wake, *n =* 185; wake to NREM, *n =* 214; NREM to REM, *n =* 96; REM to wake, *n =* 37). Lower panel: the mean calcium transients averaged from the upper traces expressed as mean (blue trace) ± SEM (shaded). Data represent means ± SEM. *P < 0.05, **P < 0.01, and ***P < 0.001.

To investigate the real-time population activity of NAc^CRH^ neurons across spontaneous sleep-wake states in free-moving mice, we recorded calcium signals of NAc^CRH^ neurons via fiber photometry. We unilaterally injected the Cre-dependent adeno-associated virus (AAV), AAV-hysn-EF1α-DIO-GCamp7f into the NAc of CRH Cre mice (Fig. 1A–C). Fiber optic probes and EEG/EMG electrodes were chronically implanted in the mice housed in their homecages to enable signal collection (Fig. 1B). Calcium fluorescence of NAc^CRH^ neurons was higher during wakefulness (10.7% ± 1.1%) than during REM (3.1% ± 0.9%) or NREM sleep (− 5.6% ± 1.0%) (Fig. 1D and E). Notably, we observed that NAc^CRH^ neurons began to increase their activities before transitions from NREM sleep to wakefulness, NREM sleep to REM sleep, and REM sleep to wakefulness, while their activity decreased before transitions from wakefulness to NREM sleep (Fig. 1F–I). These findings suggest that NAc^CRH^ neurons regulate natural sleep-wake states.

### Activation of NAc^CRH^ Neurons Promote Wakefulness and Inhibited NREM Sleep

**Fig. 2:**
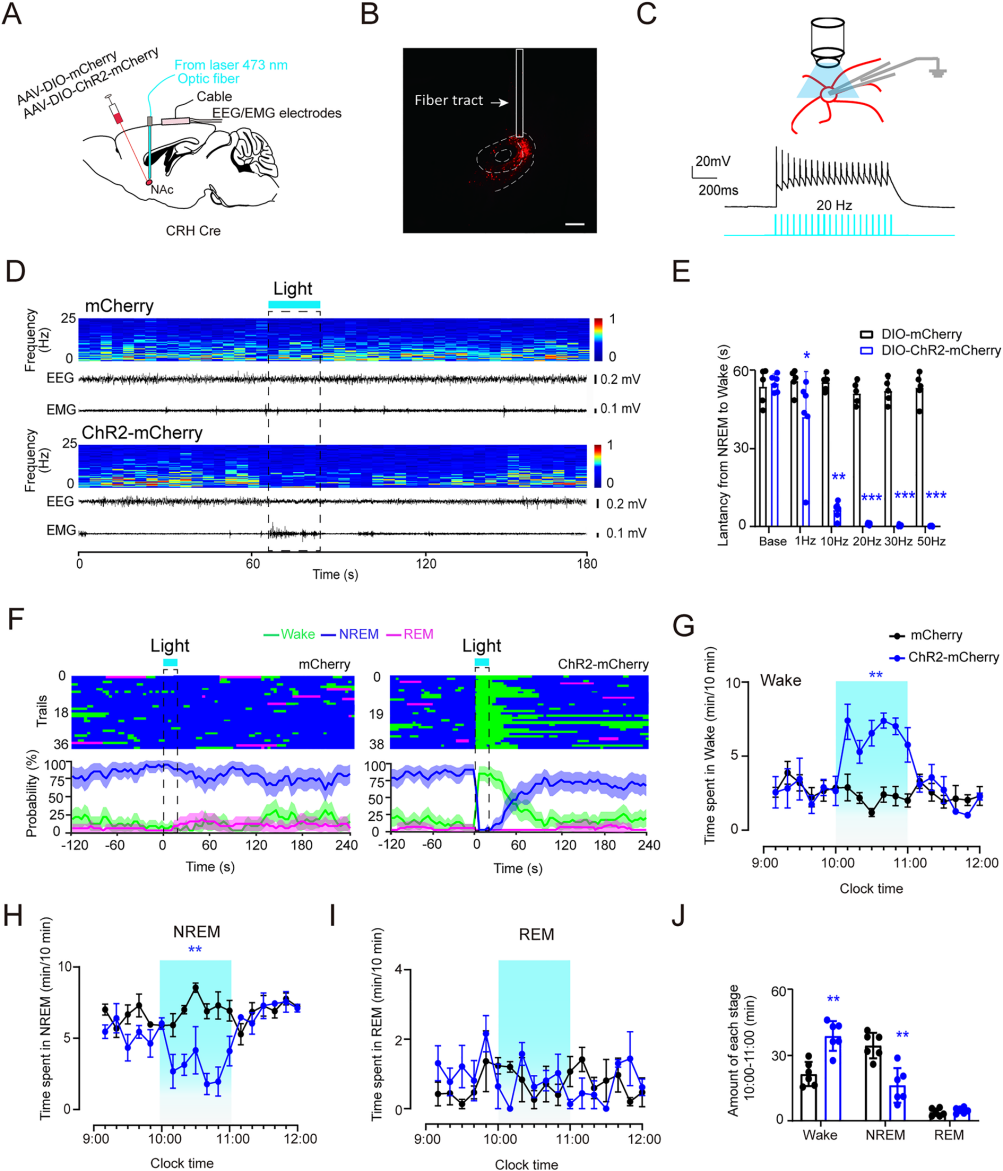
Optogenetic activation of NAc^CRH^ neurons promotes wakefulness. **A** Schematic illustration of optogenetic activation of NAc^CRH^ neurons. **B** Example image showing expression of ChR2-mCherry in NAc^CRH^ neurons. Scale bar, 250 µm. **C** Example whole-cell recording from ChR2-expressing NAc^CRH^ neurons in slice preparation showing membrane depolarization in response to light stimulation (473 nm, 20 Hz, 5 ms pulses for 1 s). **D** Example recordings of EEG/EMG traces and heat map of EEG power spectrum showing that laser stimulation (5 ms pulses at 20 Hz, for 20 s) of NAc^CRH^ neurons induced a shift from NREM to wakefulness in the mouse transfected with mCherry ChR2 (lower panel), but not with mCherry (upper panel), in NAc^CRH^ neurons. **E** Changes in transition latency from NREM sleep to wakefulness in response to different frequencies of light stimulation of the NAc^CRH^ neurons transfected with ChR2 (*n =* 6 per group, unpaired *t-test*; Base, *t*_10_ = 0.7, *P* = 0.9; 1 Hz, *t*_10_ = 1.3, *P* = 0.3; 10 Hz, *t*_10_ = 4.9, *P* = 4 × 10^−4^; 20 Hz, t_10_ = 21.4, *P* = 4 × 10^−7^; 30 Hz, *t*_10_ = 17.3, *P* = 6× 10^−8^; 50 Hz, *t*_10_ = 24.6, *P *= 1 × 10^−10^). **F** Example recordings of sleep-wake state changes (top) and the averaged probabilities of sleep-wake states (bottom) in mCherry (left) and ChR2-mCherry (right) mice. Blue bars indicate light delivery, (5 ms pulses at 20 Hz for 20 s). **G–I** Summarized time course changes of wakefulness, NREM sleep, and REM sleep profiles in response to 1 h optogenetic stimulation (blue shadow) of the NAc^CRH^ neurons transfected with mCherry (black circles) or ChR2-mCherry (blue circles) (*n =* 6, repeated-measures ANOVA; *F*_1,9_ = 10.5, *P*(Wake) = 0.004, *P*(NREM) = 0.006, *P*(REM) = 0.52). **J** Summarized total time spent in each state during the light stimulation (10:00–11:00) in mCherry (black) or ChR2-mCherry (blue) (Total amounts of each stage in control and photostimulation groups (*n =* 6, paired *t-test*; *t*_4_ = 2.1 (Wake), *t*_4_ = 3.9 (NREM), 5.2 (REM); *P*(Wake) = 0.002, *P*(NREM) = 0.003, *P*(REM) = 0.21. Data represent means ± SEM. *P < 0.05, **P < 0.01.

To directly examine the causal relationship between NAc^CRH^ neuron activation and sleep-wake behavior, we used optogenetics to specifically activate NAc^CRH^ neurons with high temporal resolution. To do this, we bilaterally injected AAV-DIO-ChR2-mCherry or AAV-DIO-mCherry into the NAc in CRH-Cre mice (Fig. 2A and B). Using whole-cell recording in brain slices, we confirmed that light stimulation (473 nm, 5 ms pulses, 20 Hz) evoked high-fidelity action potential firings in ChR2-positive NAc^CRH^ neurons (Fig. 2C). We next applied a light stimulation at 20 Hz for 20 s* in vivo* through an optical fiber on NAc^CRH^ neurons in CRH-Cre mice during NREM sleep. We found that the light stimulation induced a time-locked shift from NREM sleep to wakefulness in mice transfected with ChR2, but not with mCherry, in NAc^CRH^ neurons (Fig. 2D and F). The shift latency from NREM sleep to wakefulness, was shortened in a stimulation-frequency-dependent manner (Fig. 2E). Moreover, we also found that optogenetic stimulation of NAc^CRH^ neurons during REM sleep induced wakefulness transitions (Fig. S1). The wakefulness was maintained as long as the light stimulation was sustained, as shown with 1 h light stimulation during the light period (10:00–11:00) (Fig. 2G–J). These data indicate that activation of NAc^CRH^ neurons was sufficient to induce and maintain behavioral wakefulness.

**Fig. 3:**
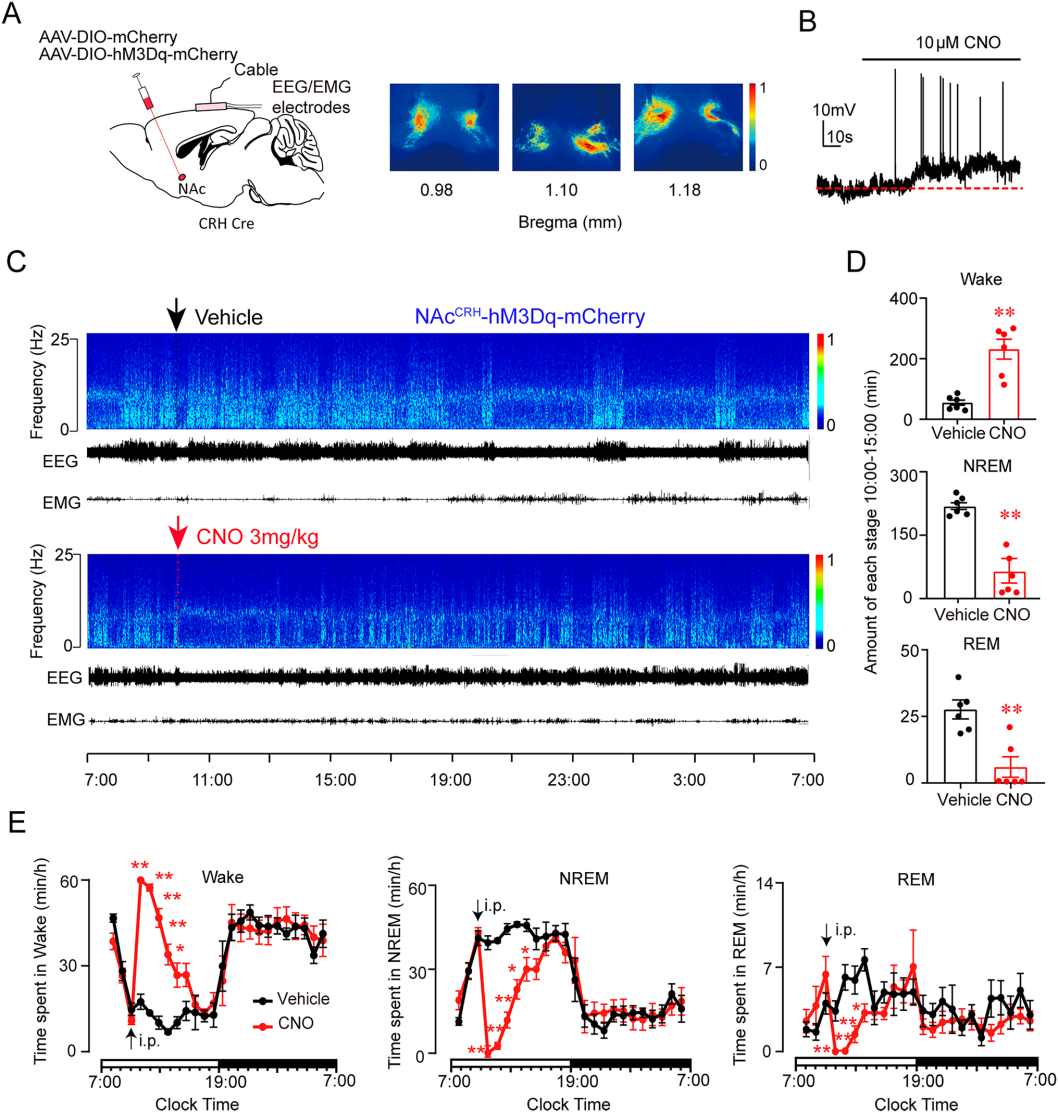
Chemogenetic activation of NAc^CRH^ neurons promotes arousal. **A** Left, schematic of virus (AAV-hSyn-DIO-hM3Dq-mCherry or AAV-hSyn-DIO-mCherry) injection into the NAc of CRH-Cre mice. Right, heatmaps show the overlay of hM3Dq expression from bregma at 0.98 mm to 1.18 mm in the NAc of all injected mice (“1” = the area of maximum overlap (red color), and “0” = the area of minimum overlap (blue color). **B** Representative whole-cell recording of hM3Dq-expressing NAc^CRH^ neurons in slice preparations showing perfusion of CNO (10 μmol/L) induced membrane depolarization and firing responses. **C** Examples of a relative EEG power, heatmap, and EEG/EMG traces, after vehicle (top) or CNO (bottom, 3mg/kg) injection at 10:00 in an hM3Dq CRH-Cre mouse. **D** The total time of each stage spent during the 5-h period (10:00–15:00) after administration of vehicle or CNO (*n =* 6, paired *t-test*). **E** Time-course changes in wakefulness, NREM sleep, and REM sleep after administration (i.p., indicated by the arrow) of vehicle or CNO in mice transfected with hM3Dq in NAc^CRH^ neurons (*n =* 6, repeated-measures ANOVA; *F*_1,12_ = 16.4 (Wake), *F*_1,12_ = 12.7 (NREM), *F*_1,12_ = 10.4 (REM); *P*(Wake) = 0.001, *P*(NREM) = 0.002, *P*(REM) = 0.004). Data represent means ± SEM. *P < 0.05, **P < 0.01.

To further examine the effect of prolonged activation of NAc^CRH^ neurons on sleep-wake behavior, we chemogenetically activated NAc^CRH^ neurons with excitatory Gq DREADD receptor, by injecting AAV-DIO-hM3Dq-mCherry or AAV-DIO-mCherry into NAc of CRH-Cre mice (Fig. 3A). Whole-cell recordings from NAc^CRH^ neurons expressing hM3Dq in brain slices showed a depolarized membrane potential and increased firing rates of the action potentials in response to perfusion of 10 μmol/L clozapine-N-oxide (CNO), a specific hM3Dq ligand (Fig. 3B). Chemogenetic activation of NAc^CRH^ neurons in CRH-Cre mice during the inactive period (daytime) induced a significant increase in wakefulness and a decrease in NREM and REM sleep for about 5 h following CNO injection at 10:00 (Fig. 3C–E). Application of CNO during the dark period, when mice are highly arousal, also significantly increases wakefulness (Fig. S2).

### Chemogenetic Inhibition of NAc^CRH^ Neurons Decreased Wakefulness and Increased NREM Sleep

**Fig. 4:**
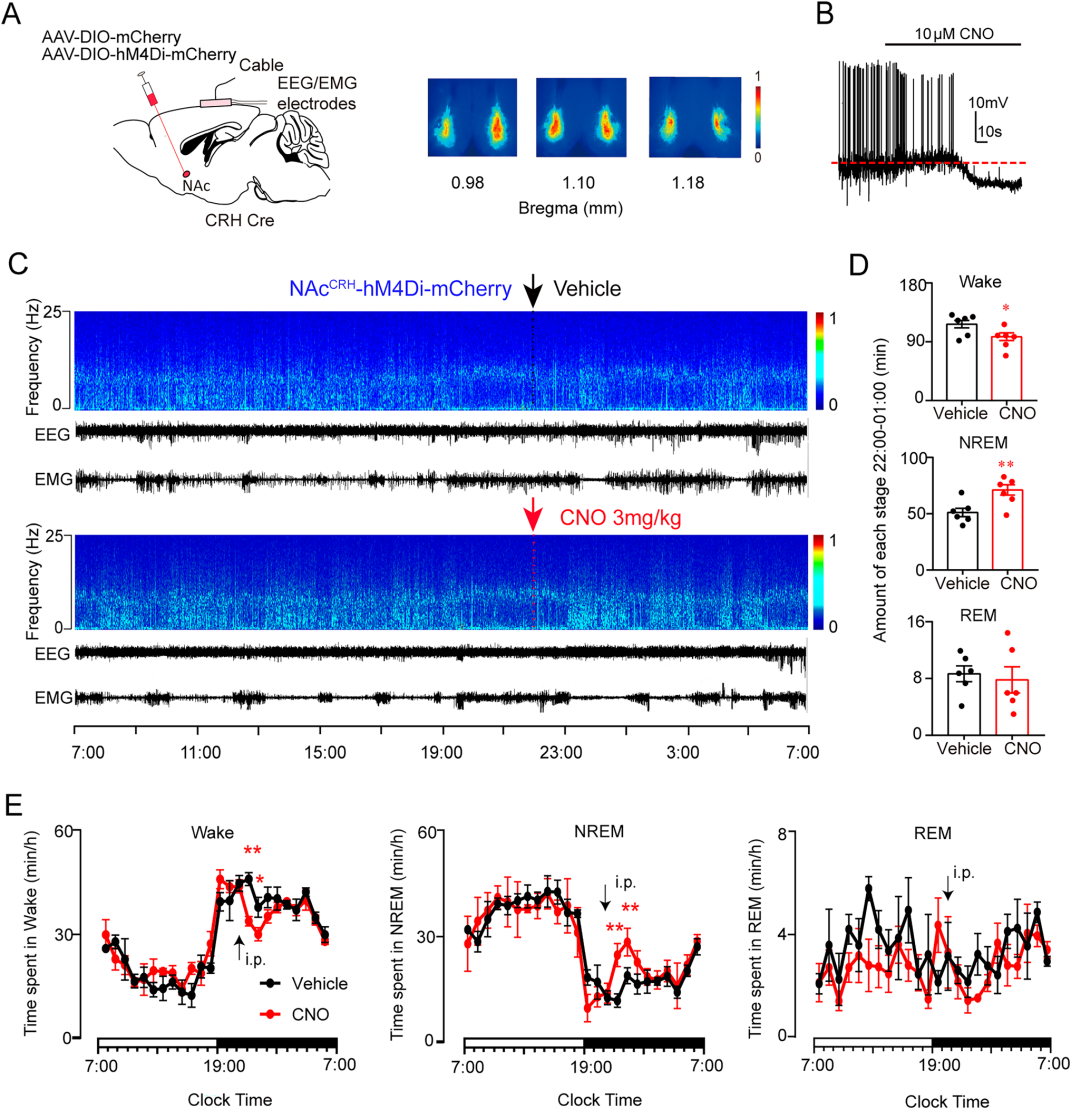
Chemogenetic inhibition of NAc^CRH^ neurons increases NREM sleep.** A** Left, schematic of bilateral injection of AAV-hSyn-DIO-hM4Di-mCherry into the NAc of CRH-Cre mice. Right, heatmaps show the overlay of hM4Di expression from bregma at 0.98 mm to 1.18 mm in the NAc of all the six injected mice (“1” = the area of maximum overlap (red color), and “0” = the area of minimum overlap (blue color). **B** Representative whole-cell recording in brain slice preparation showing that perfusion of CNO (10 μmol/L) induced hyperpolarization and reduced firing responses in NAc^CRH^ neurons transfected with hM4Di. **C** Example recording showing the time course of a relative EEG power, heatmap and EEG/EMG traces in response to a vehicle (top) or CNO (bottom) injection at 22:00 in a CRH-Cre mouse transfected with hM4Di in NAc. **D** The total amount of each sleep-wake stage spent during the 3-h period (22:00–01:00) after administration of vehicle or CNO (*n =* 6, paired *t-test*). **E** Summarized time-course changes in wakefulness, NREM sleep, and REM sleep in response to ip application (marked by arrows) of vehicle (black circles) or CNO (red circles) in mice transfected with hM4Di in NAc^CRH^ neurons (*n =* 6, repeated-measures ANOVA; *F*_1,17_ = 21.7 (Wake), *F*_1,17_ = 19.4 (NREM), *F*_1,17_ = 14.1 (REM); *P*(Wake) = 0.004, *P*(NREM) = 0.007, *P*(REM) = 0.12). Data represent means ± SEM. *P < 0.05, **P < 0.01.

To examine whether the tonic activity of NAc^CRH^ neurons affects wakefulness, we chemogenetically inhibited NAc^CRH^ neurons by injecting an AAV encoding modified inhibitory muscarinic M4 receptors (AAV-DIO-hM4Di-mCherry) or vehicle (AAV-DIO-mCherry) into the NAc in CRH-Cre mice (Fig. 4A). Whole-cell recordings from brain slices showed that CNO application of (10 μmol/L) induced apparent membrane hyperpolarization and decreased action potentials firing in hM4Di-positive NAc^CRH^ neurons (Fig. 4B). Chemogenetic inhibition of NAc^CRH^ neurons in CRH-Cre mice during the active period (dark time) induced a significant decrease in wakefulness and an increase in NREM for about 3 h following CNO administration at 22:00 (Fig. 4C–E). However, CNO injection at 10:00 (inactive period) did not significantly affect NREM sleep and wakefulness, as compared with vehicle controls (Fig. S3), possibly due to the fact that the activity of NAc^CRH^ neurons is already very low during the inactive period (Fig. 1D–F) so that the effects of chemogenetic inhibition is masked.

### Selective Activation and Inactivation of NAc^CRH^ Neurons Bidirectionally Regulates Emotional Behaviors

**Fig. 5:**
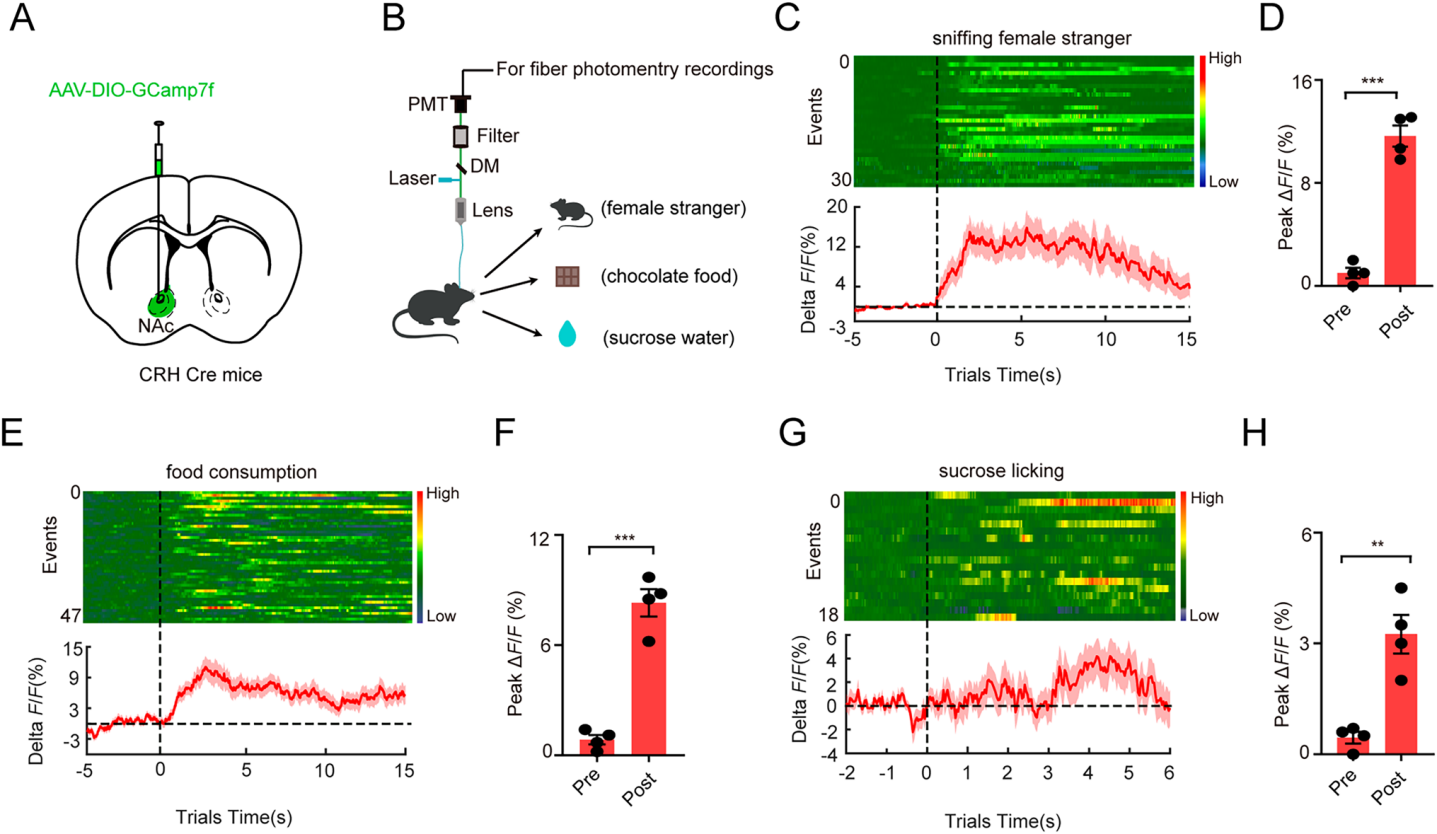
Rewarding stimuli induce activation of NAc^CRH^ neurons.** A** Schematic of stereotaxic delivery of AAV-DIO-GCamP7f injected into the NAc of CRH-Cre mice. **B** Schematic of the recording setup and* in vivo* fiber photometry recording configuration. (**C**, **E**, and **G**) Heat map (Top) and plot (Bottom) responses aligned to the onset of sniffing of the female stranger (**C**), food consumption (**E**), and sucrose licking (**G**). In the social interaction task, when mice sniffed the female stranger Ca^2+^ signal was recorded. In the food consumption task when mice were chewing pellets, a Ca^2+^ signal was recorded. In the sucrose licking task when mice were licking Ca^2+^ signal was recorded. **D** Peak Δ*F/F* calcium response during sniffing of the female stranger (*n =* 30 bouts, four mice, two-tailed unpaired t-test with Welch’s correction, *P* = 0.0007) and averaged activity of 5 s pre-sniffing compared to post-sniffing initiation over the testing period. **F** Peak Δ*F*/*F* calcium response during food consumption (*n* = 47 bouts, four mice, two-tailed unpaired *t*-test with Welch’s correction, *P* = 0.0007) and averaged activity of 5 s pre-consumption food compared to post-consumption food initiation over the testing period. **H** Peak Δ*F*/*F* calcium response during sucrose licking (*n* = 18 bouts, four mice, two-tailed unpaired t-test with Welch’s correction, *P* = 0.0045) and averaged activity of 2 s pre-licking sucrose compared to post-licking sucrose initiation over the testing period. All data represent means ± SEM. *P < 0.05, **P < 0.01, and ***P < 0.001.

The NAc is critical in reward-seeking and emotional processing, brain functions that operate in a high arousal state. We further investigated how NAc^CRH^ neurons change their activity in response to various salient reward stimuli involving sexual social, appetitive, and sucrose licking. To do this, we transfected NAc^CRH^ neurons with AAV-DIO-GCamP7f into the NAc of the CRH-Cre mice (Fig. 5A and B). Social interaction with a female stranger is a natural reward to male adults. We found that during sexual social behavioral tests, sniffing the female stranger increased calcium activity in NAc^CRH^ neurons (Fig. 5C and D). Consistent with the result of sexual social, increased GCaMP fluorescence in NAc^CRH^ neurons was detected during chocolate consumption and sucrose liking test (Fig. 5E–H). These data indicate that natural reward stimuli induce activation of NAc^CRH^ neurons.

**Fig. 6:**
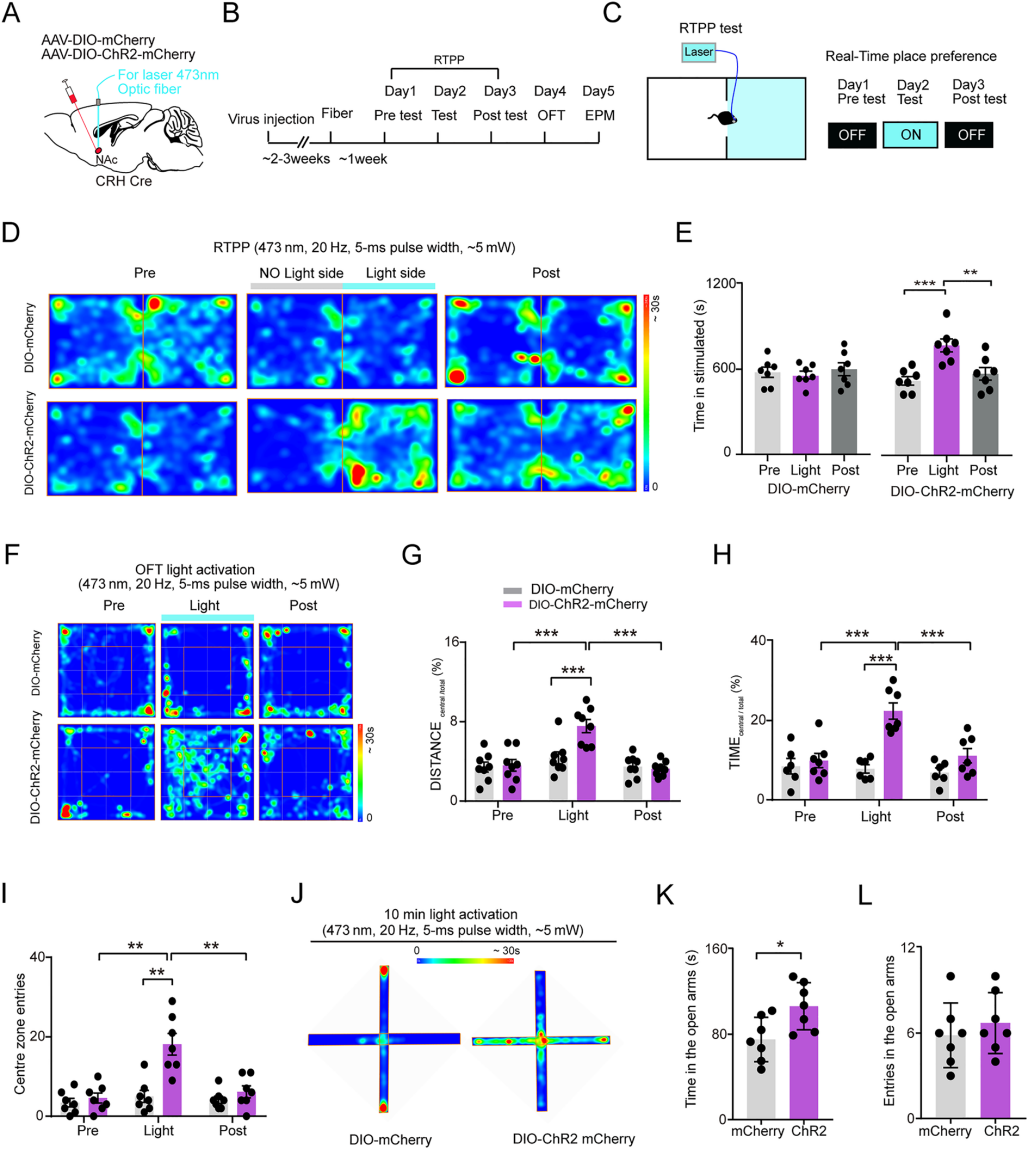
Optogenetic activation of NAc^CRH^ neurons decreased basal anxiety level. **A** Schematic of stereotaxic delivery of AAV-DIO-ChR2-mCherry or AAV-DIO-mCherry injected into the NAc of CRH-Cre mice. **B** Experimental process of the photostimulation-evoked behavior test. **C** Schematic of the real-time place preference (RTPP) test. (**D–E**) Representative heatmap and quantification of the time spent in the stimulated chamber in the RTPP test before (Pre), during (Light), and after (Post) blue laser illumination (473 nm, 20 Hz, 5-ms pulse width, ~5 mW) of the NAc transfected with mCherry or ChR2 in CRH-Cre mice (mCherry-NAc^CRH^: Kruskal-Wallis test, *P* = 0.8793; Pre vs. Light: *P *= 0.9671, Light vs. Post: *P* = 0.7632; ChR2-NAc^CRH^: Kruskal-Wallis test, *P* = 0.0005; Pre vs. Light: *P* = 0.0004, Light vs. Post:* P* = 0.0017). **F** Representative tracks of the open field test (OFT) with optogenetic activation (473 nm, 20 Hz, 5-ms pulse width, ~5 mW) of NAc^CRH^ neurons in CRH-Cre mice (Pre, Light, and Post; 5 min for each phase). **G**–**I** Quantification of the total distance traveled in the central area **G** (two-way ANOVA, Pre: *P* = 0.0100, Light: *P* = 0.0005, Post: *P* = 0.0125, interaction: *P* = 0.0019; *post hoc* Tukey’s test, Pre (mCherry vs. ChR2-mCherry): *P* = 0.0301; Light (mCherry vs. ChR2-mCherry): *P* = 0.0008; Post (mCherry vs. ChR2-mCherry): *P* = 0.0148; ChR2-mCherry (Pre vs. Light): *P* = 0.0001; ChR2-mCherry (Light vs. Post):* P* = 0.0002), time spent in the central zone **H** (two-way ANOVA, Pre: *P* = 0.0324, Light:* P* = 0.0003, Post: *P* = 0.0225, interaction: *P* = 0.0065; *post hoc* Tukey’s test, Pre(mCherry vs. ChR2-mCherry): *P* = 0.0642; Light (mCherry vs. ChR2-mCherry): *P* = 0.0003; Post (mCherry vs. ChR2-mCherry): *P* = 0.0673; ChR2-mCherry (Pre vs. Light): *P* = 0.0005; ChR2-mCherry (Light vs. Post): *P* = 0.0004), the number of central zone entries **I** (two-way ANOVA, Pre: *P* = 0.0144, Light: *P* = 0.0033, Post: *P* = 0.0455, interaction: *P* = 0.034; *post hoc* Tukey’s test, Pre (mCherry vs. ChR2-mCherry): *P* = 0.0942; Light (mCherry vs. ChR2-mCherry): *P *= 0.004; Post (mCherry vs. ChR2-mCherry): *P* = 0.0774; ChR2-mCherry (Pre vs. Light):* P* = 0.003; ChR2-mCherry (Light vs. Post): *P* = 0.006) in the OFT before (Pre), during (Light), and after (Post) 15-min illumination of the NAc in CRH-Cre mice transfected with AAV-DIO-mCherry or AAV-DIO-ChR2-mCherry. **J**–**L** Example exploration heatmap in EPM (**J**), quantification of time spent (**K**) (two-tailed unpaired t-test with Welch’s correction, *P* = 0.045) and entries (**L**) (two-tailed unpaired t-test with Welch’s correction, *P* = 0.328) in the open arms of the elevated plus maze (EPM) test with 10 min optogenetic activation (473 nm, 20 Hz, 5-ms pulse width, ~5 mW) of mCherry mice (left) and ChR2-mCherry mice (right). Data represent means ± SEM. *P < 0.05, **P < 0.01 and ***P < 0.001.

We then examined whether the wake-promoting NAc^CRH^ neurons are also involved in the regulation of emotional behavior. To do this, we transfected NAc^CRH^ neurons with AAV-DIO-ChR2-mCherry or vehicle AAV-DIO-mCherry in CRH-Cre mice (Fig. 6A). The real-time place preference (RTPP) assay (Fig. 6B and C) showed that CRH-ChR2-mCherry mice with photostimulation of NAc^CRH^ neurons spent significantly more time in the stimulated chamber, even during the post-photostimulation period (Fig. 6D and E). We next used an open-field test (OFT) and elevated plus maze (EPM) to examine the roles of NAc^CRH^ neurons in anxiety-like behavior. We found 5 min optogenetic activation of CRH-ChR2-mCherry mice significantly increased travel distance in the central area exploration time (Fig. 6F–H) and center zone entries compared with CRH-Cre mice (Fig. 6F–I), indicating hyperactivity and decreased basal anxiety level. Consistently, optogenetic activation of NAc^CRH^ neurons in CRH-Cre mice significantly increased the time spent in the EPM open arms (Fig. 6K), but no significant differences were observed in the number of open-arm entries (Fig. 6L).

Next, we examined the effects of chemogenetic inhibition of NAc^CRH^ neurons on emotional behaviors in CRH-Cre mice transfected with AAV-DIO-hM4Di-mCherry (Fig. S4A–S4B). We found that CNO treatments induced a conditioned place aversion behavior in hM4Di-mCherry mice, which spent significantly less time in the CNO treatment side chamber (Fig. S4C–S4E). Chemogenetic inhibition of NAc^CRH^ neurons in hM4Di-mCherry mice also robustly decreased exploration time in the central area of OFT (Fig. S4F) and in the EPM open arms (Fig. S4G). Together, these data indicate that activation of NAc^CRH^ neurons induces a reward-seeking behavior and a decreased basal anxiety level, whereas inactivation of NAc^CRH^ neurons results in an aversion response and an increased anxiety level in mice.

The major projection neurons in NAc are medium spiny neurons (MSNs), which account for ~ 90%–95% of all NAc neurons and can be further classified into D1R-MSNs and D2R-MSNs respectively [24]. We performed fluorescence in situ hybridization analysis for mRNA localization using RNAscope and found that the percentage of NAc^CRH^ neurons expressing *Drd1* and *Drd2* was ~ 67.51% and ~ 25.66% respectively, whereas ~ 33.28% of NAc^CRH^ neurons express neither *Drd1* nor *Drd2.* Interestingly, ~ 21.35% of NAc^CRH^ neurons co-expressed with both *Drd1* and *Drd2*, indicating that most *Drd2-*expressing NAc^CRH^ neurons also express *Drd1*. In other words, ~ 4.59% of NAc^CRH^ neurons expressing *Drd2* alone, in contrast to ~ 46.21% of NAc^CRH^ neurons expressing *Drd1* alone (Fig. S5A–S5B)*.* On the other hand, the percentage of D1R-MSNs and D2R-MSNs neurons expressing *Crh* was ~ 31.14% and ~ 29.13% respectively (Fig. S5C–S5D). We found that ~ 98.5% of NAc^CRH^ neurons expressing Vgat (*Slc32a1*, a vesicular GABA transporter), confirming that the majority of NAc^CRH^ neurons are GABAergic (Fig. S5E–S5F).

### Identification of Direct Synaptic Innervation from NAc^CRH^ Neurons to BNST Neurons

To determine the downstream circuit of NAc^CRH^ neurons inducing arousal and anxiolytic behavior, we first labeled NAc^CRH^ axons and presynaptic terminals by injecting Adeno-Associated Virus (AAV)-DIO-mGFP-T2A-Synaptophysin-mRuby (Red fluorescent protein) into the NAc of CRH-Cre mice (Fig. S6A–S6B). We found that NAc^CRH^ neuronal terminals are densely distributed in several brain areas including the bed nucleus of stria terminals (BNST), the ventral tegmental area (VTA), the lateral hypothalamus (LH) and the substantia nigra pars reticulata (SNr) (Fig. S6C–S6D).

**Fig. 7:**
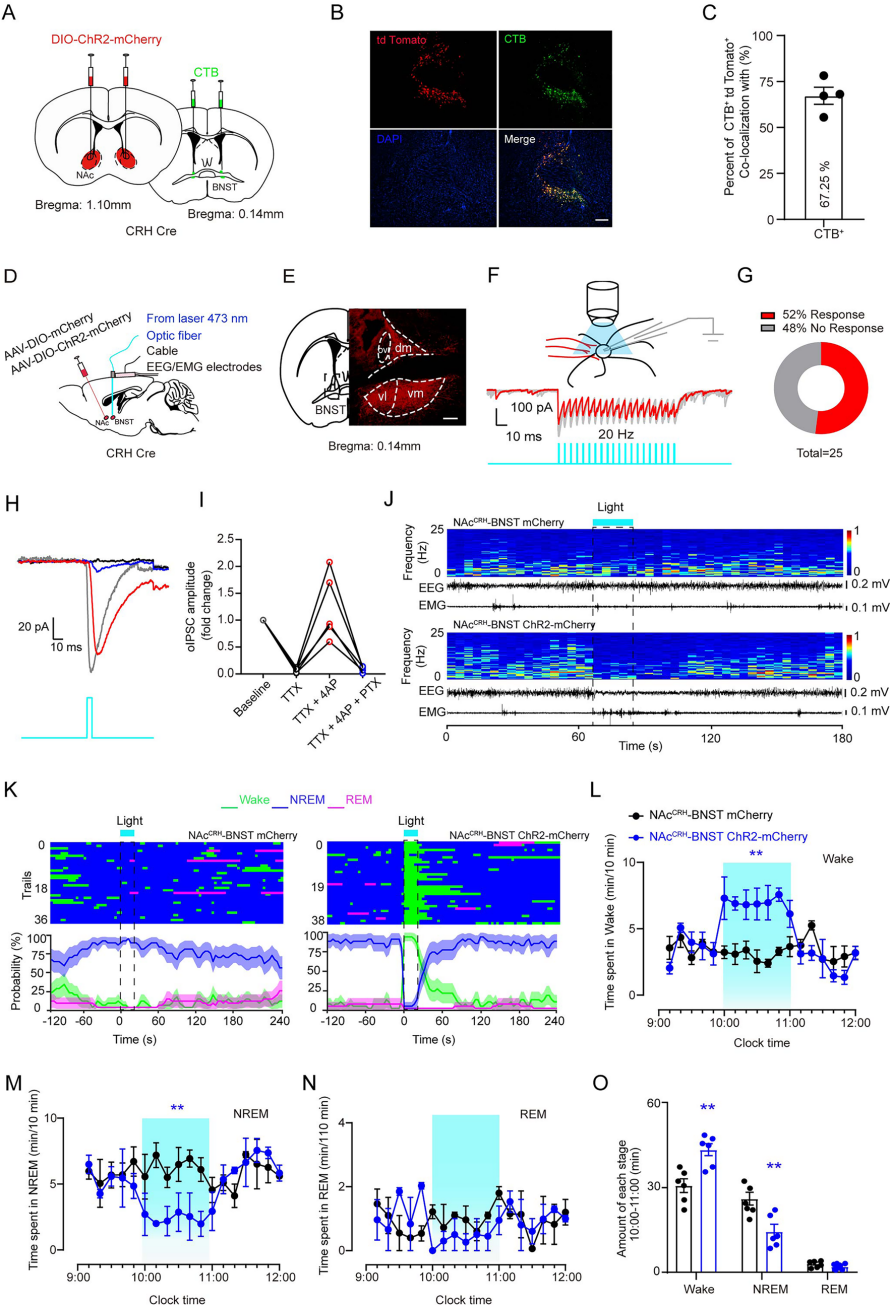
Optogenetic stimulation of the NAc^CRH^-BNST pathway induces arousal. **A** Schematic of experimental design showing injection of AAV-DIO-ChR2-mCherry virus into the NAc and CTB into the BNST of the CRH-Cre mice. **B** Example confocal images showing expression of AAV-DIO-ChR2-mCherry (red) and CTB (green) in the NAc. Scale bar, 100 μm. **C** Quantification of CTB and tdTomato (CRH neurons) overlap in the NAc. Percentage of CTB^+^ and tdTomato^+^ cells in CTB^+^ cells of the NAc (3 slices per mouse,* n* = 4). **D** Diagram showing injection of AAV-DIO-mCherry or AAV-DIO-ChR2-mCherry into the NAc, and implantation of optical fibers into the bed nucleus of stria terminals (BNST) in CRH-Cre mice. **E** Representative images of NAc^CRH^ neuronal fibers expressing ChR2-mCherry at the BNST. scale bar, 100 μm. **F** Typical traces of inhibitory postsynaptic currents (IPSCs) recorded from BNST neurons in brain slice preparations evoked by laser stimulation of ChR2-expressing NAc^CRH^ neuronal terminals in the BNST. **G** Proportion of recorded BNST neurons that responded to laser stimulation of ChR2-expressing NAc^CRH^ neuronal terminals in the BNST. **H**, **I** Example traces (**H)** and quantification (**I)** of oIPSCs, which were abolished by bath application of TTX and reintroduced by TTX together with 4-AP, and then were completely abolished by application of TTX together with 4-AP and PTX. **J** Example recordings of EEG/EMG traces and heat map of EEG power spectrum showing that laser stimulation (5 ms pulses at 20 Hz for 20 s) of NAc^CRH^ neuronal terminals in BNST induced a shift from NREM to wakefulness in the mouse transfected with mCherry ChR2 (lower panel), but not with mCherry (upper panel), in NAc^CRH^ neurons. **K** Example recordings of sleep-wake state changes (top) and the averaged probabilities of sleep-wake states (bottom) in mCherry (left) and ChR2-mCherry (right) mice. Blue bars indicate light delivery (5 ms pulses at 20 Hz for 20 s). **L–N** Summarized time course changes of wakefulness, NREM sleep, and REM sleep profiles in response to 1 h optogenetic stimulation (blue shadow) of the NAc^CRH^ neuronal terminals in BNST in mCherry (black circles) or ChR2-mCherry (blue circles) transfected mice (*n =* 6, repeated-measures ANOVA,* F*_1,16_ = 12.3 (Wake), *F*_1,16_ = 14.1 (NREM), *F*_1,16_ = 9.8 (REM); *P*(Wake) = 0.007, *P*(NREM) = 0.008, *P*(REM) = 0.42). **O** Summarized total time spent in each state during the light stimulation (10:00-11:00) in mCherry (black) or ChR2-mCherry (blue) (Total amounts of each stage in control and photostimulation groups (*n =* 6, paired *t-*test, *t*_6 _= 9.4 (Wake), *t*_6_ = 6.9 (NREM), *t*_6_ =3.2 (REM); *P*(Wake) = 0.003, *P*(NREM)= 0.006, *P*(REM) = 0.47). Data represent means ± SEM. *P < 0.05, **P < 0.01.

The BNST acts as a relay station between cortical limbic regions and a critical node in the social behavioral network, as well as an arousal-promoting area [25]. We then hypothesize that NAc^CRH^ neurons modulate arousal and emotional behaviors through the downstream target BNST. To further characterize NAc-BNST connections, we injected AAV-DIO-ChR2-mCherry into the NAc and injected cholera-toxin subunit B (CTB) into the BNST to retrogradely label NAc neurons projecting to BNST (Fig. 7A). We found that ~ 67.25% of CTB-labeled cells in the NAc were tdTomato^+^ cells (Fig. 7B and C), indicating that major BNST-projecting neurons in NAc are NAc^CRH^ neurons.

We further verified the subtypes of NAc neurons projecting to BNST by injecting retrograde tracer (Red Retrobeads, tdRBs) in the BNST and carried out triple fluorescence in situ hybridization in NAc (Fig. S7A and S7B). When examined by co-staining of tdRB cells with *Crh* and *Drd1* probes we found that among retrogradely labeled tdRB^+^ cells in NAc, ~ 75.25% of cells expressed with *Crh,* including cells expressed with *Crh* only (*Crh*^*+*^* Drd1*^*−*^, ~ 9.46%) and cells co-expressed with both *Crh* and *Drd1 (Crh*^*+*^* Drd1*^*+*^, ~ 65.8%). In addition, we found that ~ 22.41% of cells expressed with *Drd1* only (*Crh*^*-*^* Drd1*^*+*^) and ~ 2.34% expressed with neither *Crh* nor *Drd1 (Crh*^*−*^* Drd1*^*−*^) (Fig. S7C). We further analyzed the co-localization of *Crh* and *Drd2* probes with tdRB^+^ cells (Fig. S7D) and found that among retrogradely labeled tdRB^+^ cells in NAc, ~ 82.21% of cells expressed with *Crh,* including cells expressed with *Crh* only (*Crh*^*+*^* Drd2*^*−*^, ~ 78.52%) and cells co-expressed with both *Crh* and *Drd2 (Crh*^*+*^* Drd2*^*+*^, ~ 3.70%*)*. In addition, we found that ~ 3.03% of cells expressed with *Drd2* only (*Crh*^*−*^* Drd1*^*+*^) and ~ 16.49% expressed with neither *Crh* nor *Drd2* (*Crh*^*−*^* Drd2*^*−*^) (Fig. S7E). It is worth noting that among retrogradely labeled tdRB^+^ *Crh*^*+*^ cells in NAc, ~ 88.23% co-expressed with *Drd1*^*+*^ and only ~ 4.51% co-expressed with *Drd2*^*+*^ (Fig. S7F). These results suggest that the major NAc projection neurons to BNST are NAc^CRH^ neurons expressing *Drd1*.

To identify the functional synaptic connections of the NAc^CRH^-BNST pathway, we injected AAV-DIO-ChR2-mCherry or AAV-DIO-mCherry into the NAc (Fig. 7D) and observed that mCherry terminals abundantly distributed in most area of BNST, but not in the ova region of the BNST (Fig. 7E). Then, we did whole-cell recordings in BNST neurons and optogenetic stimulated NAc^CRH^ axon terminals in BNST in brain slices (Fig. 7F). We found that light stimulation evoked inhibitory postsynaptic currents (oIPSCs) in ~ 52% of neurons recorded (Fig. 7G). The oIPSCs were abolished in the presence of tetrodotoxin (TTX, 1 μmol/L) and were rescued by perfusion with a K^+^ channel blocker 4-aminopyridine (4-AP, 100 μmol/L). The rescued oIPSP was completely abolished by application of a GABA_A_ receptor blocker picrotoxin (PTX, 100 μmol/L) (Fig. 7H and I). These results indicate that about half of BNST neurons receive direct GABAergic synaptic innervation from NAc^CRH^ neurons forming GABAergic monosynaptic.

### Activation of Axon Terminals of NAc^CRH^ Neurons in BNST Promotes Wakefulness and Positive Emotional Behaviors

We next examined whether direct activation of the NAc^CRH^-BNST pathway similarly regulates sleep-wake and emotional behaviors. We found that brief photostimulation (20 Hz for 20 s) of NAc^CRH^ neuronal terminals in the BNST induced an immediate transition from NREM sleep to wakefulness (Fig. 7J and K). The time course of the quantified sleep-wake period showed that 1 h photostimulation of NAc^CRH^-BNST projection terminals applied from 10:00 to 11:00 induced a significant increase in wakefulness and a decrease in NREM sleep (Fig. 7L–O). Notably, we also found that optogenetic stimulation of the NAc^CRH^-BNST pathway induced wakefulness during REM sleep (Fig. S8). These results suggest that the effects of NAc^CRH^ neuron activation on arousal are through the downstream target BNST.

**Fig. 8:**
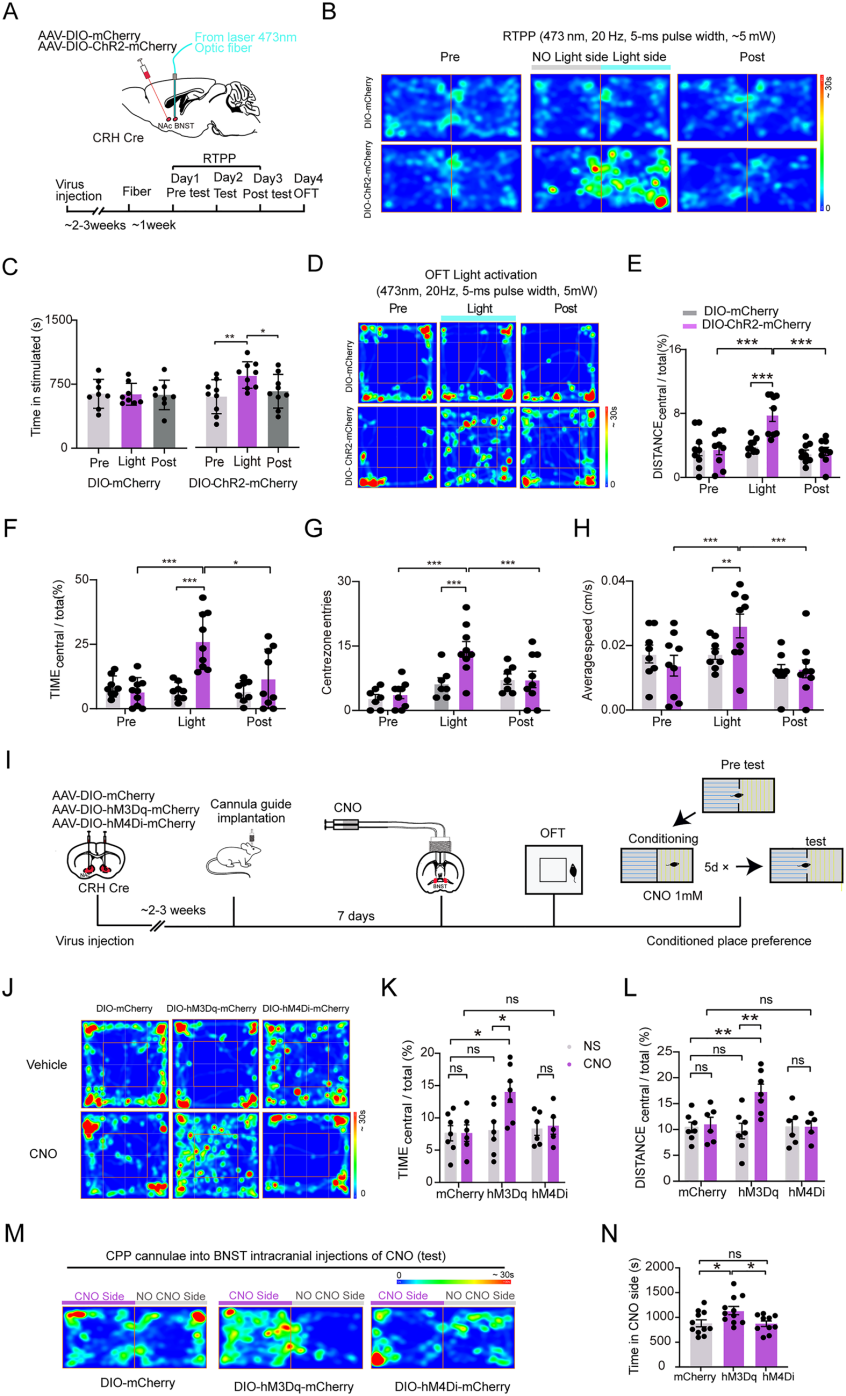
Effects of optogenetic activation of NAc^CRH^-BNST pathway on emotional behaviors. **A** Top, diagram showing injection of AAV-DIO-mCherry or AAV-DIO-ChR2-mCherry into the NAc, and implantation of optical fibers into BNST in CRH-Cre mice. Bottom, experimental process of the photostimulation-evoked behavior test. **B**, **C** Representative heatmap (**B)** and quantification (**C)** of the time spent in the stimulated chamber in the RTPP test before (Pre), during (Light), and after (Post) laser illumination (473 nm, 20 Hz, 5-ms pulse width, ~5 mW) of the NAc^CRH^ neuronal terminals in BNST in mCherry or ChR2-mCherry mice (mCherry-NAc^CRH^-BNST: Kruskal-Wallis test, *P* = 0.9745; Pre vs. Light: *P *= 0.9141, Light vs. Post: *P *= 0.8962; ChR2-NAc^CRH^-BNST: Kruskal-Wallis test, *P* = 0.003; Pre vs. Light: *P* = 0.0035, Light vs. Post: *P* = 0.02). **D** Heatmap illustration of the OFT with optogenetic activation (473 nm, 20 Hz, 5-ms pulse width, ~5 mW,) of the NAc^CRH^ neuronal terminals in BNST in CRH-Cre mice transfected with mCherry (top) or ChR2-mCherry (bottom) in NAc^CRH^ neurons (Pre, Light, and Post; 5 min for each phase). **E**–**H** Quantification of the total distance traveled in the central area **E** (two-way ANOVA, Pre: *P* = 0.1473, Light: *P* = 0.0005, Post: *P *= 0.2416, interaction: *P* = 0.0142; *post hoc* Tukey’s test, Pre (mCherry vs. ChR2-mCherry): *P *= 0.7316; Light (mCherry vs. ChR2-mCherry): *P* = 0.0001; Post (mCherry vs. ChR2-mCherry): *P *= 0.6423; ChR2-mCherry (Pre vs. Light):* P* = 0.0003, ChR2-mCherry (Light vs. Post): *P* = 0.0002), time spent in the central zone **F** (two-way ANOVA, Pre: *P *= 0.1104, Light: *P* = 0.0003, Post:* P* = 0.0216, interaction: *P* = 0.0032; *post hoc* Tukey’s test, Pre (mCherry vs. ChR2-mCherry): *P* = 0.3316, Light (mCherry vs. ChR2-mCherry): *P* = 0.0004, Post (mCherry vs. ChR2-mCherry): *P *= 0.4133, ChR2-mCherry (Pre vs. Light): *P* = 0.0002, ChR2-mCherry (Light vs. Post): *P* = 0.0321), the number of central zone entries **G** (two-way ANOVA, Pre: *P *= 0.3265, Light: *P* = 0.0004, Post: *P* = 0.0455, interaction: *P* = 0.012; *post hoc* Tukey’s test, Pre (mCherry vs. ChR2-mCherry): *P* = 0.2549; Light (mCherry vs. ChR2-mCherry): *P *= 0.0003; Post (mCherry vs. ChR2-mCherry): *P* = 0.4774; ChR2-mCherry (Pre vs. Light):* P* = 0.0004; ChR2-mCherry (Light vs. Post): *P* = 0.002) and the average speed **H** (two-way ANOVA, Pre: *P *= 0.3231, Light: *P* = 0.0049, Post: *P *= 0.5457, interaction: *P* = 0.041; *post hoc* Tukey’s test, Pre (mCherry vs. ChR2-mCherry): *P* = 0.3942; Light (mCherry vs. ChR2-mCherry): *P* = 0.0072; Post (mCherry vs. ChR2-mCherry): *P* = 0.4774; ChR2-mCherry (Pre vs. Light): *P* = 0.0003, ChR2-mCherry (Light vs. Post): *P* = 0.0004) in the OFT before (Pre), during (Light), and after (Post) 15-min illumination of the NAc^CRH^ neuronal terminals in BNST in CRH-Cre mice transfected with AAV-DIO-mCherry (grey) and AAV-DIO-ChR2-mCherry (pink). **I** Experimental timeline and schematic showing injection of Cre-dependent hM3Dq and mCherry virus into the NAc of a CRH-Cre mouse and chemogenetic activation of NAc^CRH^ terminals in BNST for behavioral tests. **J** Representative heatmap in the OFT test. **K** Percentage of time spent in the central zone of OFT (two-way ANOVA, mCherry(NS vs. CNO): *P* = 0.1420, hM3Dq (NS vs. CNO): *P* = 0.0373, hM4Di (NS vs. CNO): *P* = 0.3341; CNO(mCherry vs. hM3Dq): *P* = 0.0224; CNO (mCherry vs. hM4Di): *P* = 0.2726, *post hoc* Tukey’s test). **L** Percentage of distance traveled in the central area of OFT (two-way ANOVA, mCherry (NS vs. CNO): *P *= 0.3726, hM3Dq (NS vs. CNO): *P* = 0.0044, hM4Di (NS vs. CNO): *P* = 0.2645; CNO (mCherry vs. hM3Dq): *P* = 0.0027; CNO (mCherry vs. hM4Di): *P* = 0.3224, *post hoc* Tukey’s test). **M** and** N** Representative tracks in the CPP test **M** and **N** quantification of the time spent in the CNO chamber (Kruskal-Wallis test, *P* = 0.4649, mCherry vs. hM3Dq: *P* = 0.0342, mCherry vs. hM4Di: *P* = 0.7185, hM3Dq vs. hM4Di:* P* = 0.0257). All data shown are the mean ± SEM and error bars represent SEM. *P < 0.05, **P < 0.01 and ***P < 0.001.

Next, we examined whether stimulating NAc^CRH^ neuronal projections to BNST similarly modulates emotional behaviors. To do this, we bilaterally injected the AAV-DIO-ChR2-mCherry or AAV-DIO-mCherry virus into the NAc and implanted optic fibers into the BNST in CRH-Cre mice (Fig. 8A). We found that light stimulation of NAc^CRH^ neuronal terminals in the BNST in mice transfected with AAV-DIO-ChR2-mCherry in NAc^CRH^ neurons significantly increased staying time in the stimulated side in the RTPP test (Fig. 8B and C). In the OFT test, light stimulation of NAc^CRH^ neuronal terminals in the BNST significantly increased the exploration time, travel distance in the central area of the OFT, center zone entries, and average speed (Fig. 8D–H), indicating that activation of NAc^CRH^-BNST pathway also has a positive valance and an anxiolytic effect.

The effects of activating the NAc^CRH^-BNST pathway on emotional behaviors were further confirmed by chemogenetic approaches. AAV-DIO-hM3Dq-mCherry or AAV-DIO-mCherry virus was bilaterally injected into NAc and guide cannula were implanted bilaterally in the BNST in CRH-Cre mice. Mice were tested with OFT or CPP after CNO or vehicle infusions into the BNST through the implanted cannula to activate NAc^CRH^ axonal terminals (Fig. 8I). Chemogenetic activation of NAc^CRH^-axonal terminals in BNST significantly increased exploration time and locomotor activities in the central area (Fig. 8J–L) in the OFT test, suggesting a decreased basal anxiety level. CNO treatment in these mice also significantly increased the time spent in the CNO side chamber in the CPP test (Fig. 8M and N). Taken together, these results showed that activation of the neural projection from the NAc^CRH^ neurons to the BNST has a positive valence and anxiolytic effect.

## Discussion

NAc and BNST are both located in the basal forebrain. NAc is also part of the striatum, with its projecting neurons classified into DR1 and DR2 neurons, both of which are GABAergic neurons [26–28]. When further analyzing the overlap ratio of NAc^CRH^ neurons with DR1 and DR2 neurons, we notice that about 46.21% of NAc^CRH^ neurons expressing DR1 only, less than 5% expressing DR2 only, 21.35% expressing both DR1 and DR2, and 33% expressing neither DR1 nor DR2 (Fig. S5). Interestingly, although only about 30% of DR1 or DR2 neurons expressed with *Crh* (Fig. S5C and S5D), about 70%–80% of BNST-projecting NAc neurons are *Crh*^*+*^ (Fig. 7C, Fig. S7C and S7E). Furthermore, 88.23% of NAc^CRH^ neurons projecting to the BNST are those expressing DR1(Fig. S7F), with only 4.5% expressing DR2 and less than 10% expressing neither DR1 nor DR2 (Fig. S7F). Therefore, DR1-expressing NAc^CRH^ neurons selectively constitute the major BNST-projecting neurons from NAc. Although 21.35% of NAc^CRH^ neurons express both DR1 and DR2 (Fig. S5B), these neurons do not project to the BNST.

Previous studies showed that activation of NAc D_1_R direct pathway promotes arousal, possibly through their projections to the midbrain and lateral hypothalamus to disinhibit dopamine neurons and orexin neurons [5], whereas activation of adenosine A_2A_ / D2 receptor-expressing indirect pathway in the NAc projecting to the ventral pallidum induces slow-wave sleep [6]. However, a recent study reported that NAc D_1_R regulated arousal through innervating POA neurons and modified nociceptive responses through innervating midbrain VTA neurons [29]. It was also reported that NAc D1 neurons regulated SWS, while D2 neurons regulated REM sleep, but the underlying circuit mechanisms are not clear [30]. The discrepancy results regarding the role of NAc D1 and D2 neurons in regulating sleep-waking behavior suggest that NAc D1 and D2 neurons may have sub-populations with distinct functions through their differential projection targets [31]. Our study identifies for the first time a subtype of NAc D1 neurons expressing CRH projecting to the BNST (Fig. 7 and Fig. S7). Moreover, activation of this NAc^CRH^-BNST pathway promotes arousal and positive valence responses including place preference and anxiolytic effects (Figs. 7 and 8).

CRH and CRH neurons are considered important stress-promoting elements in the brain [14, 32, 33]. Since CRH neurons have a relatively widespread distribution in the brain [34, 35], it is plausible that the exact role of CRH neurons depends on the functions of the specific brain regions they are distributed and their circuitry connections. In contrast to the effects of CRH neurons reported in other brain regions that CRH neurons usually promote stress-related arousal accompanied by negative emotional states such as anxiety [36–38], we found that the activation of the NAc^CRH^-BNST pathway promotes arousal along with positive valence effects such as place preference and anxiolysis (Figs. 7 and 8B–H). The BNST is a critical center for regulating negative emotional behaviors [39–42]. GABAergic NAc^CRH^ neurons may exert their effects by inhibiting BNST neuron activity. Whether the arousal effects of NAc^CRH^ neurons are mediated by the same or different downstream BNST subtype neurons responsible for positive emotional responses requires further investigation. It is also of interest to elucidate in future studies whether these effects of NAc^CRH^ neurons are mediated by their release of CRH, GABA, or both.

Seeking reward and avoiding harm is the basic instinctual behavior essential for animal survival. On one hand, animals must maintain high levels of arousal to react quickly in dangerous and stressful situations to avoid harm [43–45]. Prolonged stress responses, however, may lead to sleep disturbances [46–48], anxiety [49, 50], and depression [51, 52]. On the other hand, increased arousal also occurs when animals anticipate reward and experience pleasure so that a high motivation level can be maintained for approach behaviors [53, 54]. Thus, both positive and negative emotional responses are accompanied by increased levels of arousal, which, though seemingly contradictory, are actually crucial adaptive behavioral responses for biological survival. Multiple sleep-wake regulating pathways have been identified in the brain [55, 56], however, it is unclear whether changes in arousal levels induced by different types of stimuli (such as stress and reward) activate the same or different sleep-wake regulating pathways. Similarly, the association and the underlying mechanisms between different sleep-wake regulatory pathways and distinct positive and negative emotions are also unclear. The elucidation of these mechanisms is important not only for understanding the interplays between sleep-wake and emotional behaviors but also for identifying therapeutic targets for relevant diseases.

## Supplementary Information

Below is the link to the electronic supplementary material.

Supplementary file1 (PDF 2240 kb)
