# Supplementary material for: Nucleus Accumbens Corticotropin-Releasing Hormone Neurons Projecting to the Bed Nucleus of the Stria Terminalis Promote Wakefulness and Positive Affective State
Source: Neurosci Bull. 2024 Jul 9;40(11):1602–20. doi: 10.1007/s12264-024-01233-y (PMC11607243; doi:10.1007/s12264-024-01233-y)
Supplement: Supplementary file 1 — Supplementary file1 (PDF 2240 kb) [file 12264_2024_1233_MOESM1_ESM.pdf]

Supplemental Figures and Legends

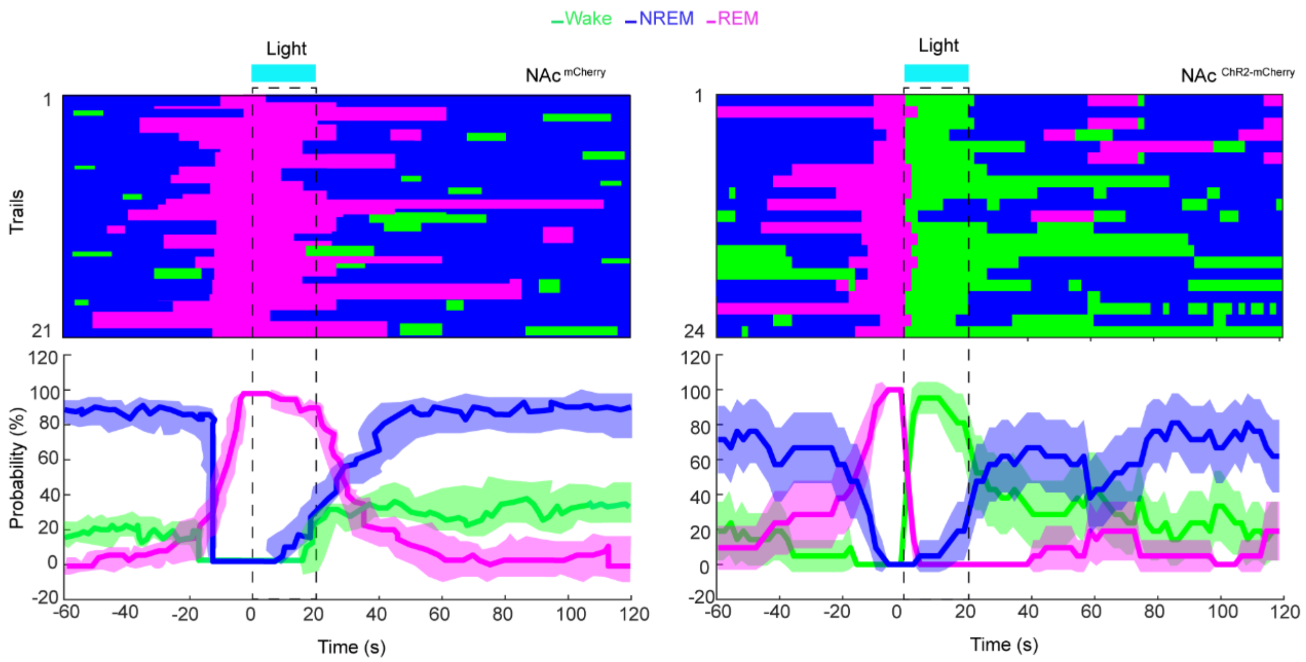

Fig. S1 Optogenetic stimulation of the NAc^{CRH} neurons during REM sleep induces arousal. Example recordings of sleep-wake state changes (top) and the averaged probabilities of sleep-wake states (bottom) in mCherry (left) and ChR2-mCherry (right) mice. Blue bars indicate light delivery (5 ms pulses at 20 Hz for 20 s).

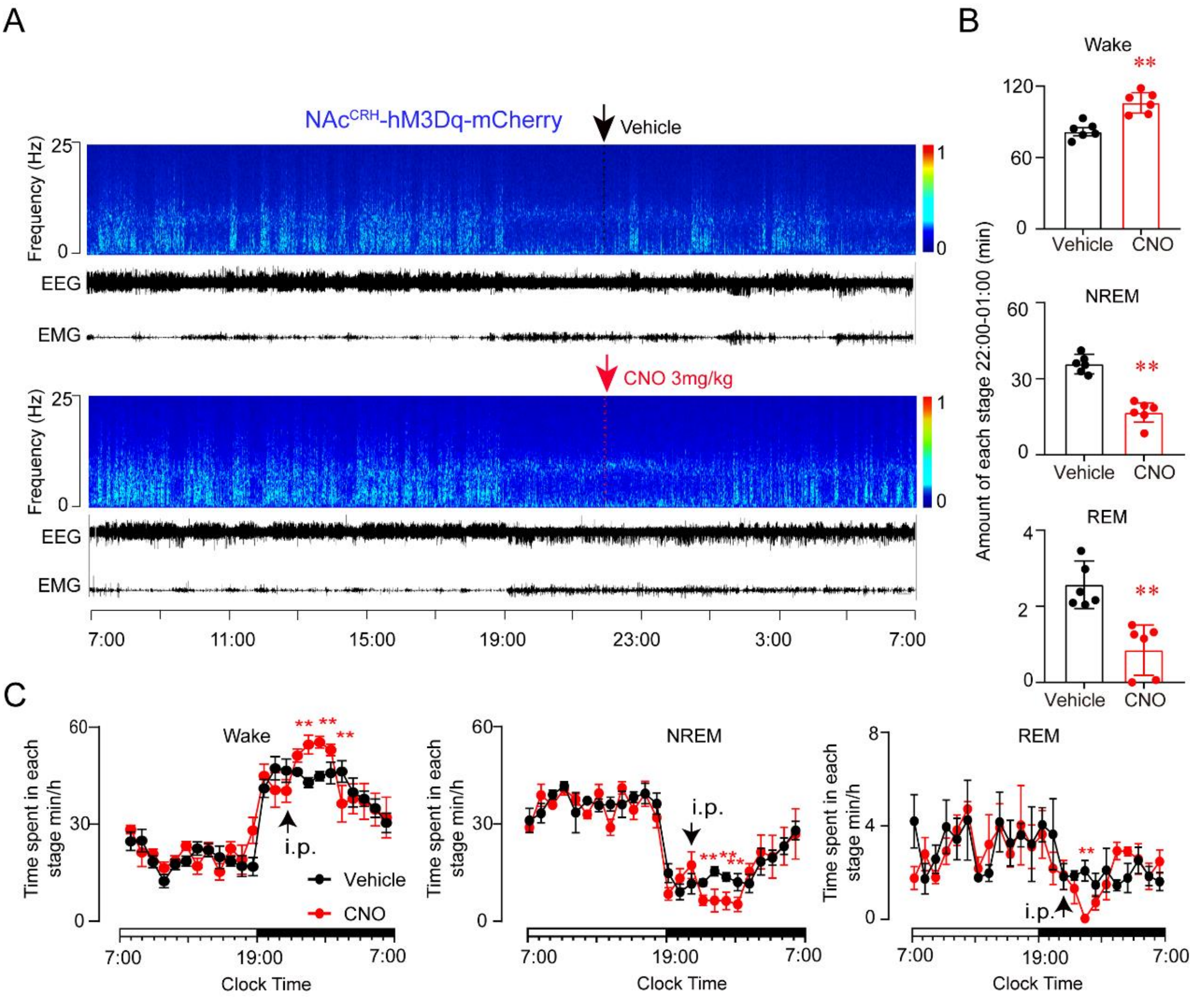

Fig. S2 Chemogenetic activation of NAc^{CRH} neurons during the dark (active) period promoted wakefulness. A Typical example of a relative EEG power, heatmap, and EEG/EMG traces, after vehicle (upper panel) or CNO (lower panel) injection at 22:00 in a CRH-Cre mouse transfected with hM3Dq in NAc. CNO = 3mg/kg. **B** The total amount of each stage spent during the 2 h period (22:00-00:00) after injection of vehicle or CNO (n = 6, paired t-test). Data represent the mean ± SEM (n = 6; *P < 0.05, **P < 0.01, using paired t-test, compared with vehicle control). **C** The time-course changes in wakefulness, NREM sleep, and REM sleep in CRH-Cre mice transfected with hM3Dq in NAc^{CRH} neurons in response to administration of vehicle or CNO (i.p., indicated by arrows). Data shown are the mean ± SEM (n = 6; *P < 0.05, **P < 0.01, using repeated-measures ANOVA, compared with vehicle control).

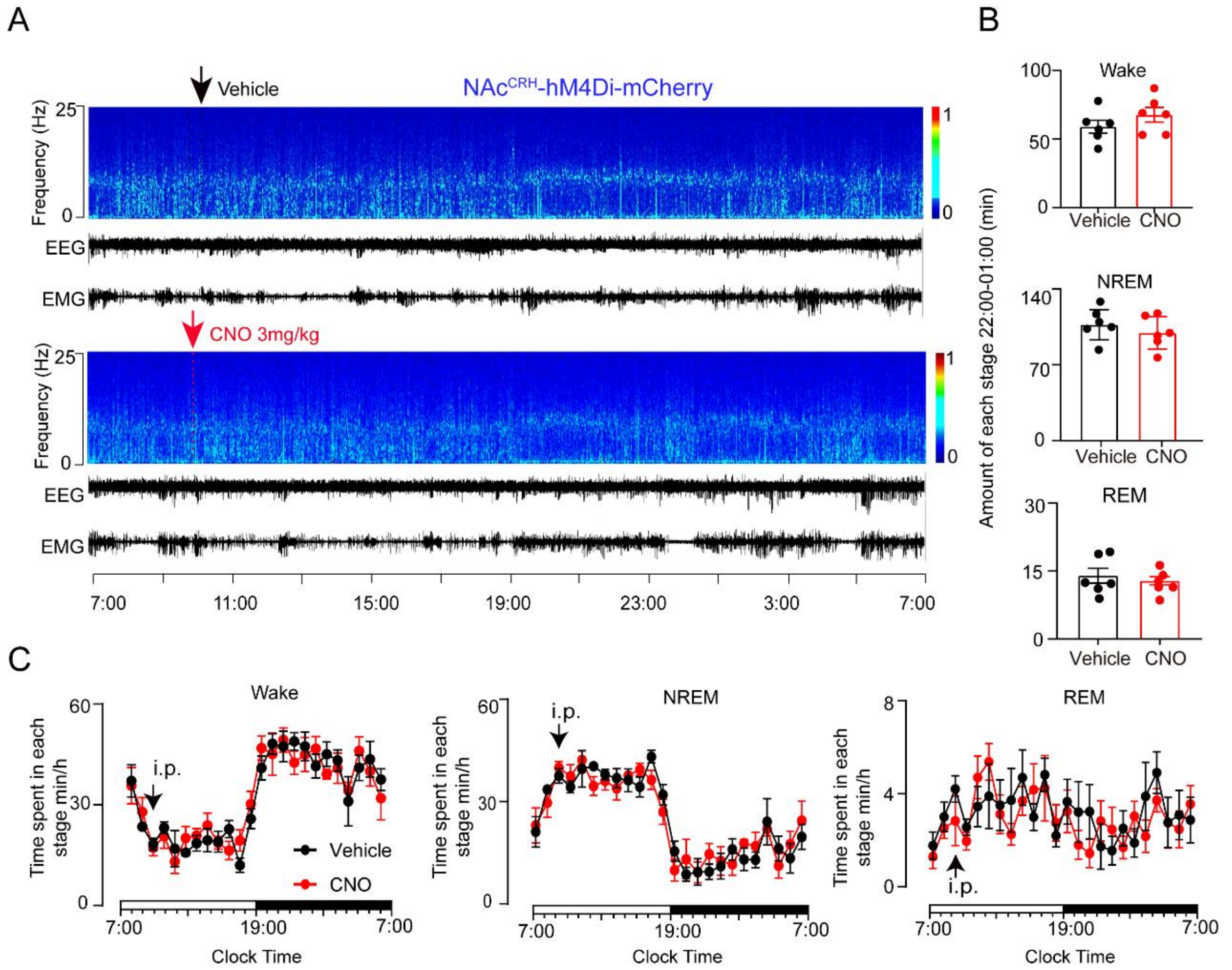

Fig. S3 Chemogenetic inhibition of NAc^{CRH} neurons did not affect the sleep-wake pattern during the light period. A Typical example of a relative EEG power, heatmap, and EEG/EMG traces, after vehicle (upper panel) or CNO (lower panel) injection at 10:00 in a CRH-Cre mouse transfected with hM4Di in NAc^{CRH} neurons. CNO = 3mg/kg. **B** The total amount of each stage spent during the 3 h period (10:00-13:00) after injection of vehicle or CNO. Data represented are the mean ± SEM (n = 6; *P < 0.05, **P < 0.01, using paired t-test, compared with vehicle control). **C** The time-course changes in wakefulness, NREM sleep, and REM sleep in CRH-Cre mice transfected with hM4Di in NAc^{CRH} neurons in response to administration of vehicle or CNO. Data represented are the mean ± SEM (n = 6; *P < 0.05, **P < 0.01, using paired t-test, compared with vehicle control).

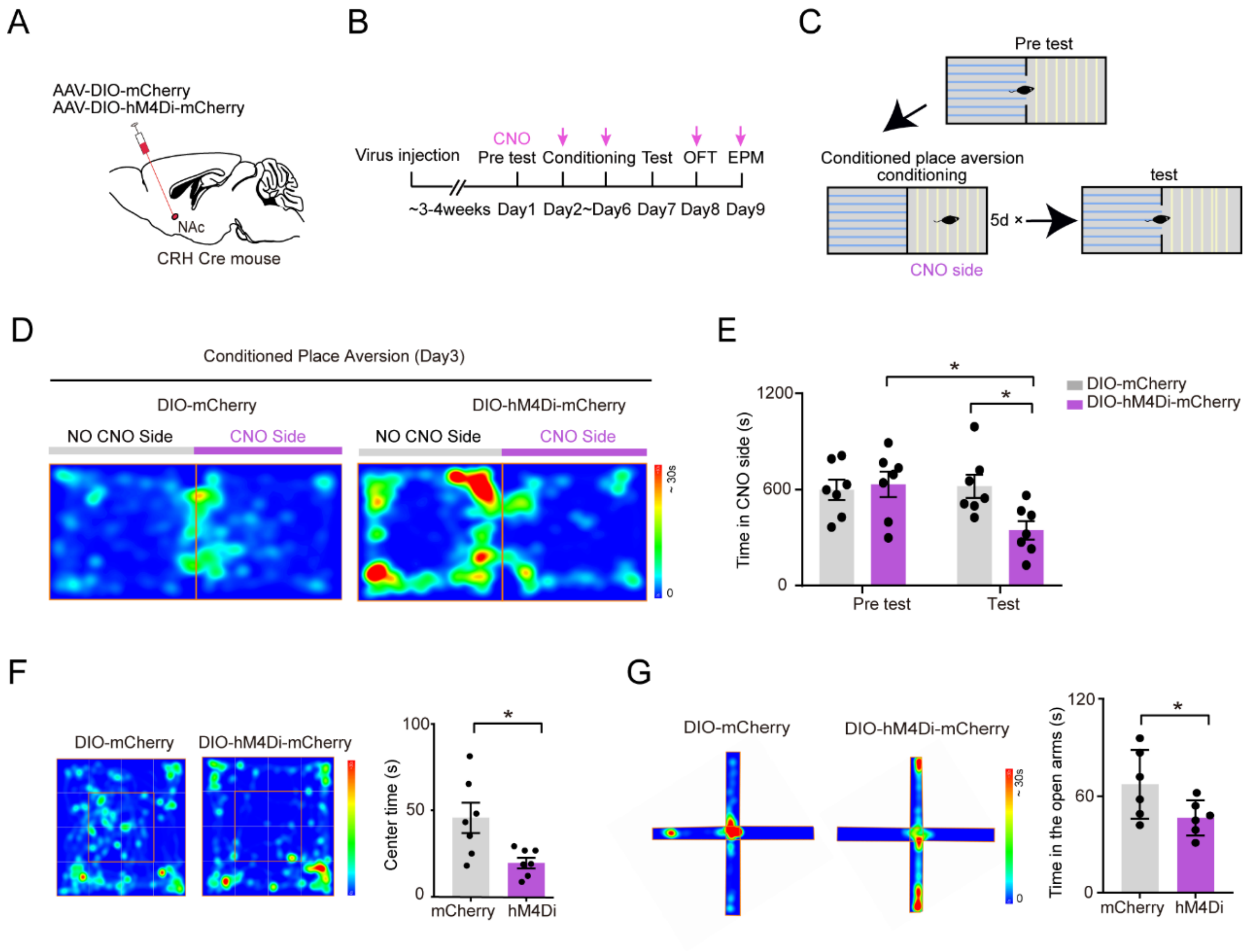

Fig. S4 Chemogenetic inhibition of NAc^{CRH} neurons increased basal anxiety level. A Schematic of stereotaxic delivery of AAV-DIO-hm4Di-mCherry or AAV-DIO-mCherry into the NAc of CRH-Cre mice. **B** Experimental process of the DREADD-hM4Di evoked behavior test. **C** Schematic of the conditioned place aversion (CPA) test. **D-E** Representative heatmap **D** and quantification of the time spent in the CNO injection (i.p., 3 mg/kg) chamber **E** in the CPA test before (Pre) and after (Post) CNO treatment in CRH-Cre mice transfected with mCherry or ChR2 in NAc. (two-way ANOVA, Pre: $P = 0.7164$, Test: $P = 0.0334$, interaction: $P = 0.0174$; post hoc Tukey's test, Pre (mCherry vs. hM4Di-mCherry): $P = 0.831$; Test (mCherry vs. hM4Di-mCherry): $P = 0.044$; hM4Di-mCherry (Pre vs. Test): $P = 0.0524$; mCherry: $n = 7$. ChR2: $n = 7$). **F** Representative locomotion heatmap (left) of the OFT and quantification of the time spent in the central zone (right) (two-tailed unpaired t-test with Welch's correction, $P = 0.021$, mCherry: $n = 7$. ChR2: $n = 7$). **G** Example exploration heatmap (left) in EPM and quantification of time spent in the open arms of EPM (right). (two-tailed unpaired t-test with Welch's correction, $P = 0.032$, mCherry: $n = 6$. ChR2: $n = 6$). All data shown are the mean ± SEM. $^*P < 0.05$, $^{**}P < 0.01$ and $^{***}P < 0.001$.

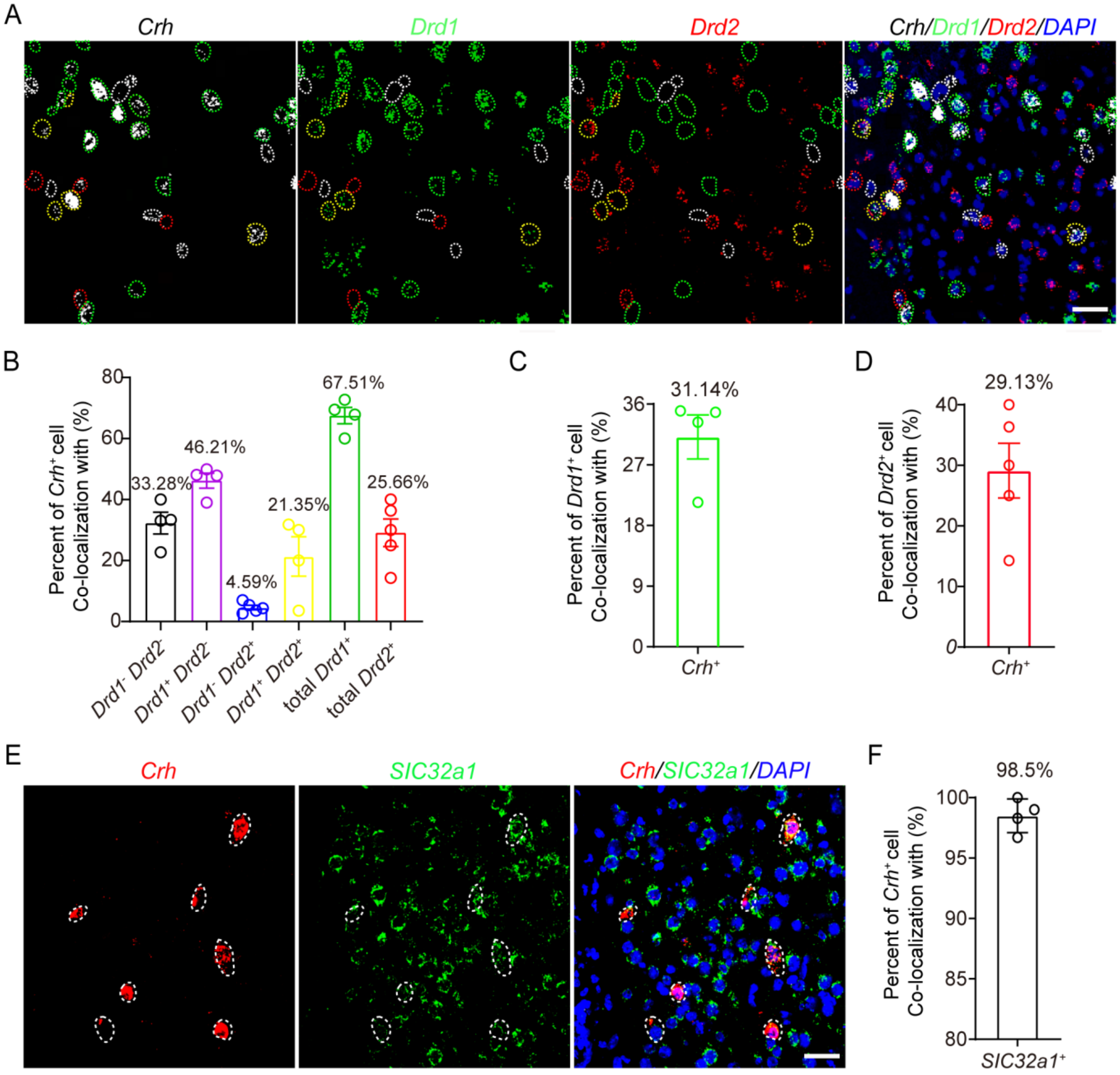

Fig. S5 Identification of CRH-expressing neurons in the NAc. A Fluorescence in situ hybridization (FISH) images in NAc with *Crh* probes (white), *Drd1* probes (green), *Drd2* probes (red), and DAPI (blue). The white dashed circles indicate *Crh*$^+$ neurons, green indicates *Drd1*$^+$ neurons, red indicates *Drd2*$^+$ neurons and yellow indicates neurons positive for all three probes (*Crh*$^+$, *Drd1*$^+$and *Drd2*$^+$). Scale bar, 100 µm. **B** Quantitative summary of *Crh*$^+$ neurons co-localized with neither *Drd1* nor *Drd2* (black column), *Drd1* only (purple column), *Drd2* only (blue column), and both *Drd1* and *Drd2* (yellow column) respectively. Total *Drd1* (green column) is the sum of *Drd1* only cells and both *Drd1* and *Drd2* cells, whereas total *Drd2* (red column) is the sum of *Drd2* only cells and both *Drd1* and *Drd2* cells. **C** and **D** Quantitative summary of *Drd1*$^+$ **C** and *Drd2*$^+$ **D** cells co-localized with *Crh*$^+$ in the NAc. **E** FISH images in NAc with *Crh* probes (red), *SIC32a1* probes (green), and DAPI (blue) in the NAc.

White dashed circles indicate *Crh*$^{+}$ cells. Scale bar, 50 μm. **F** Quantitative analyses of percent of *Crh*$^{+}$ cells co-localized with *SIC32a1*$^{+}$ in the NAc. n = 3 mice. All data are the mean ± SEM.

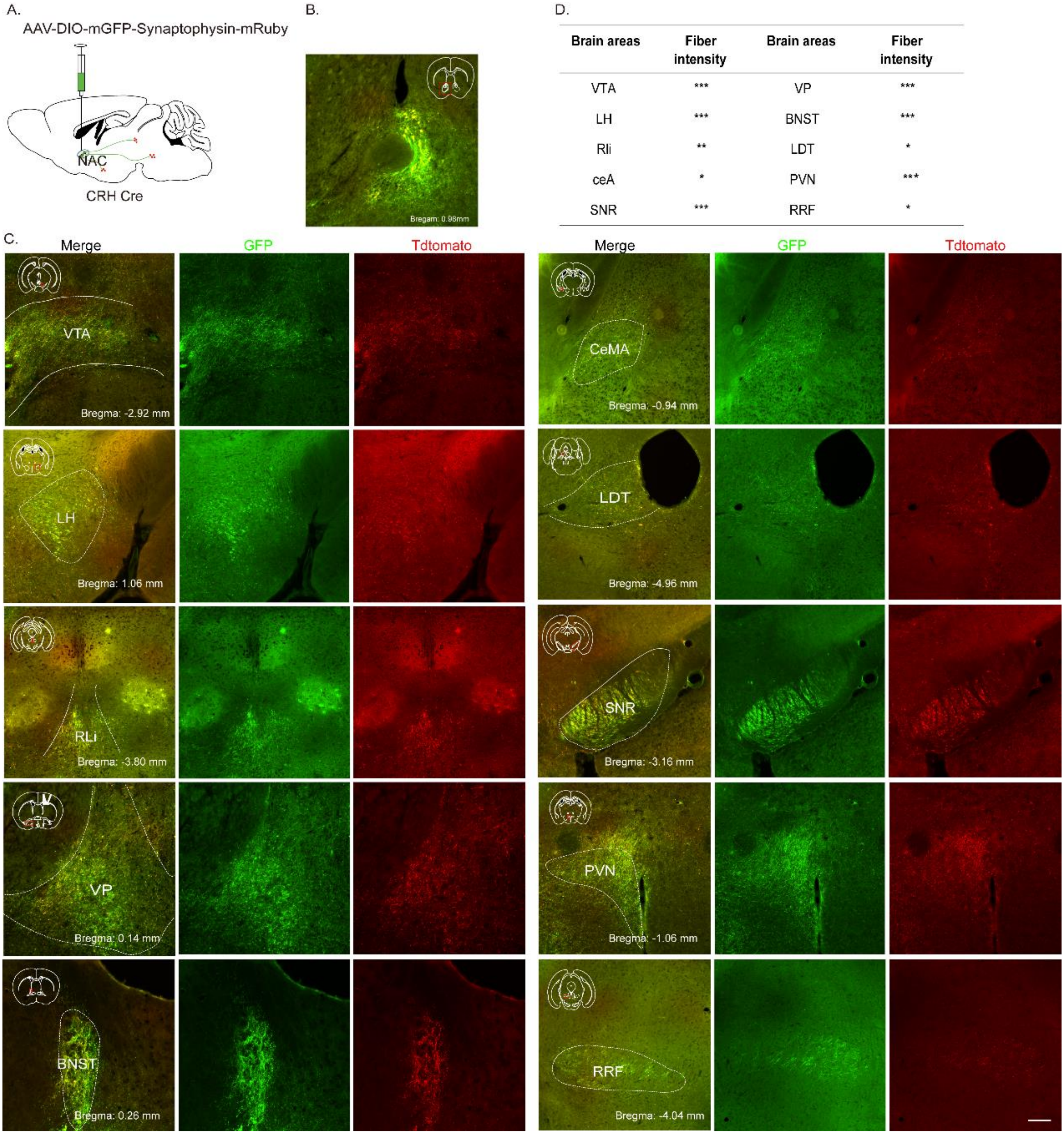

Brain areas	Fiber intensity	Brain areas	Fiber intensity
VTA	***	VP	***
LH	***	BNST	***
RIi	**	LDT	*
ceA	*	PVN	***
SNR	***	RRF	*

Fig. S6 Projection mapping of NAc^{CRH} neurons. A Schematic of stereotaxic delivery of AAV-DIO-mGFP-T2A-Synaptophysin-mRuby injected into the NAc of CRH-Cre mice. **B** Typical image showing the injection site of AAV-DIO-mGFP-T2A-Synaptophysin-mRuby in the unilateral NAc. **C** Example images of brain areas with density distribution of axons (green) and axonal terminals (red) of NAc^{CRH} neurons. Abbreviations: VTA, ventral tegmental area; LH, lateral hypothalamus; RLi, rostral linear

nucleus of the raphe; CeMA, central amygdaloid nucleus, medial division, anterior part; VP, ventral pallidum; BNST, bed nucleus of stria terminals; LDT, laterodorsal tegmental nucleus; PVN, paraventricular thalamic nucleus; SNR, substantia nigra, reticular part; RRF, retroparafascicular nucleus. **D** Semi-quantification of the density of Synaptophyin-mRuby^{+} terminals in the brain areas innervated by NAc^{CRH} neurons. * represent mean axonal terminals intensity (0–90). *: 1–30; **: 30–60; ***: 60–90.

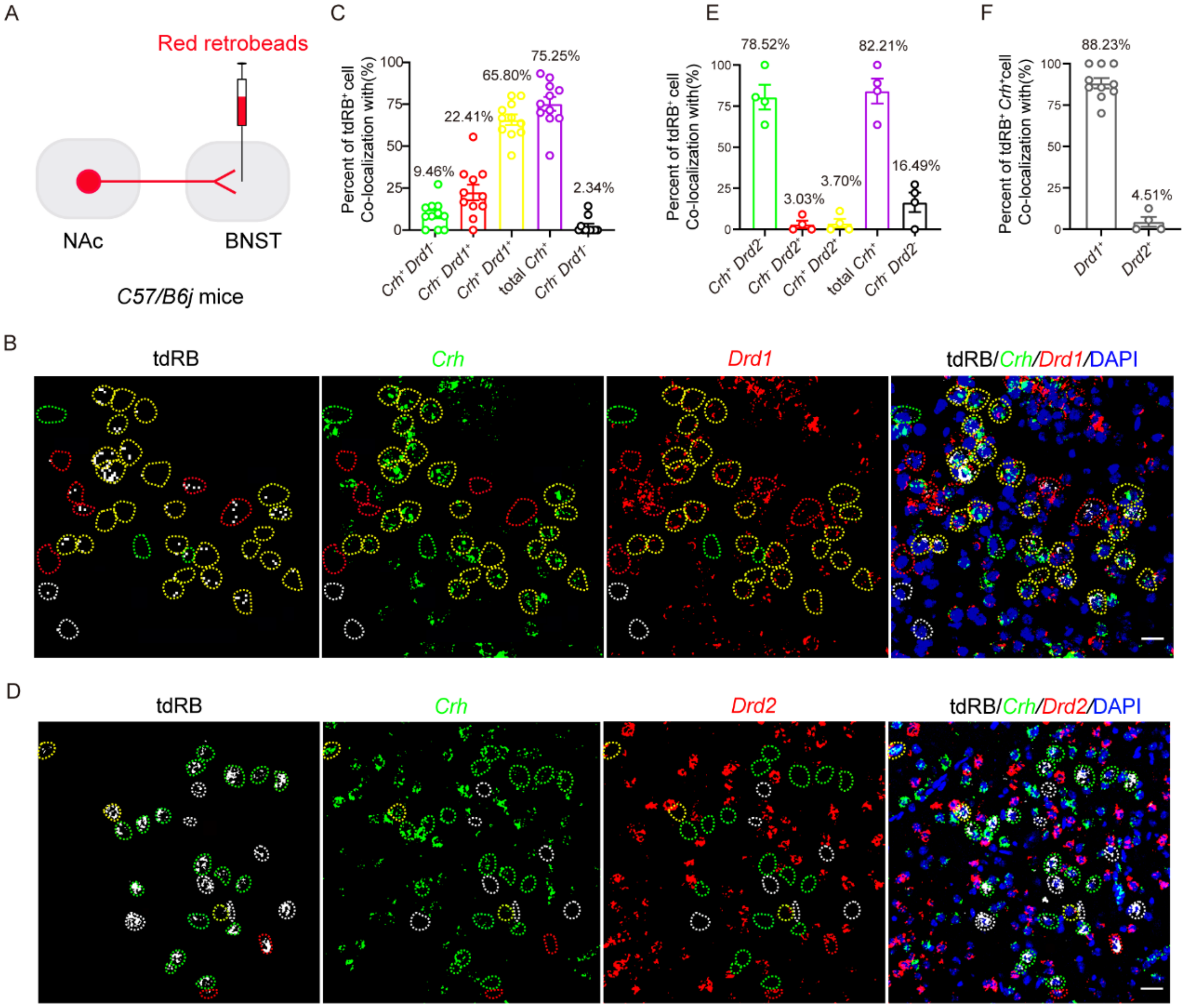

Fig. S7 Identification of NAc Neuronal subtypes projecting to BNST. A Schematic of experimental design showing labeling of NAc neurons projecting to BNST by injecting retrograde tracer (Red Retrobeads, tdRBs) in the BNST of *C57/B6j* mice. **B** FISH images in NAc co-labeled with tdRB (white), *Crh* probes (green), *Drd1* probes (red), and DAPI (blue). The different colored dashed circles indicate tdRB^{+} neurons co-localized with *Crh* only (green circles), *Drd1* only (red circles), neither *Crh*

nor *Drd1* (white circles), and both *Crh* and *Drd1*(yellow circles) respectively. Scale bar, 50 µm. **C** Quantitative analyses of tdRB$^+$ cells co-localized with *Crh* only (green column), *Drd1* only (red column), neither *Crh* nor *Drd1* (black column), and both *Crh and Drd1*(yellow column). Total *Crh* (purple column) is the percent of cells expressing *Crh* only plus that expressing both *Crh* and *Drd1* cells. **D** FISH images in NAc with tdRB (white), *Crh* probes (green), *Drd2* probes (red), and DAPI (blue). The dashed circles in different colors indicate tdRB$^+$ neurons co-localized with *Crh* only (green circles), *Drd2* only (red circles), neither *Crh* nor *Drd2* (white circles), and both *Crh* and *Drd2*(yellow circles) respectively. Scale bar, 100 µm. **E** Quantitative analyses of tdRB$^+$ cells co-localized with *Crh* only (green column), *Drd2* only (red column), neither *Crh* nor *Drd2* (black column), and both *Crh* and *Drd2* (yellow column) respectively. Total *Crh* (purple column) is the percent of cells expressing *Crh* only plus that expressing both *Crh* and *Drd2*. **F** Percent of tdRB-retro-labeled *Crh*$^+$ cells expressing *Drd1*$^+$and *Drd2*$^+$ respectively. n = 3 mice. All data are the mean ± SEM.

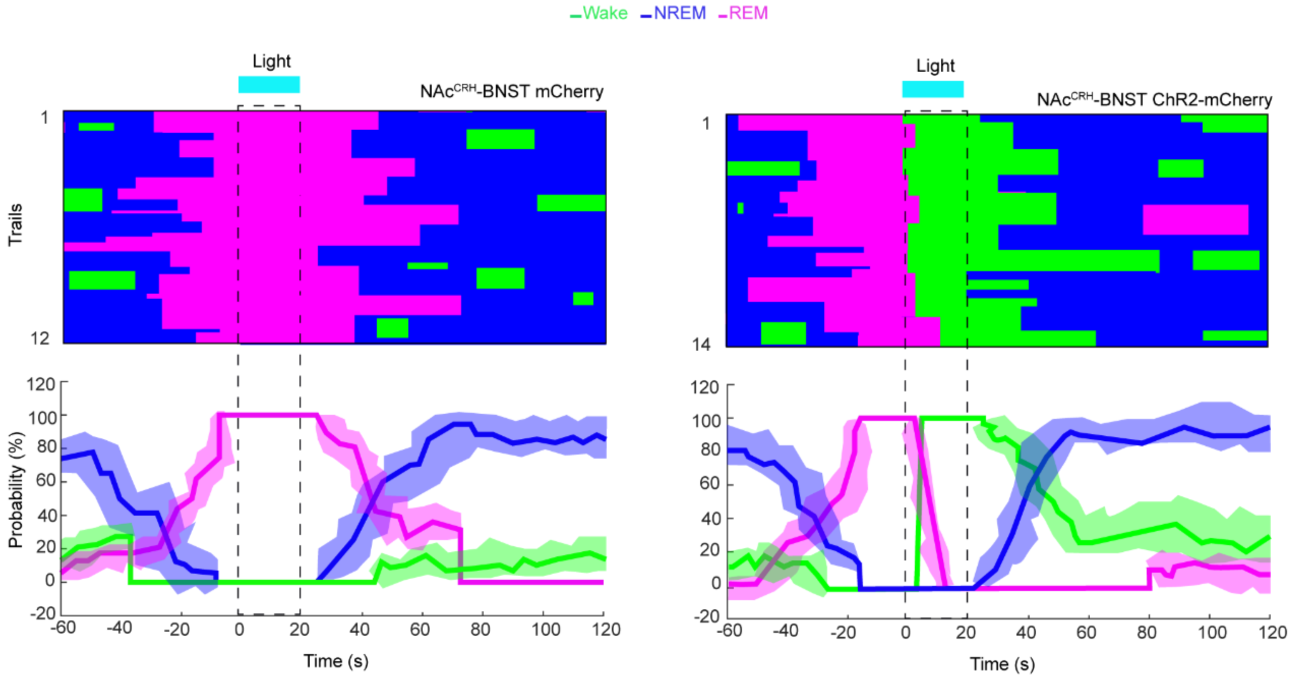

Fig. S8 Optogenetic stimulation of the NAc^{CRH}-BNST pathway during REM sleep induces arousal. Example recordings of sleep-wake state changes (top) and the averaged probabilities of sleep-wake states (bottom) in mCherry (left) and ChR2-mCherry (right) mice. Blue bars indicate light delivery (5 ms pulses at 20 Hz for 20 s).
